# Re-formulating Gehan’s design as a flexible two-stage single-arm trial

**DOI:** 10.1186/s12874-019-0659-2

**Published:** 2019-01-28

**Authors:** Michael J. Grayling, Adrian P. Mander

**Affiliations:** MRC Biostatistics Unit, University of Cambridge, Cambridge, CB2 0SR UK

**Keywords:** Adaptive, Binary, Group sequential, One-sample, Phase II, Single-arm

## Abstract

**Background:**

Gehan’s two-stage design was historically the design of choice for phase II oncology trials. One of the reasons it is less frequently used today is that it does not allow for a formal test of treatment efficacy, and therefore does not control conventional type-I and type-II error-rates.

**Methods:**

We describe how recently developed methodology for flexible two-stage single-arm trials can be used to incorporate the hypothesis test commonly associated with phase II trials in to Gehan’s design. We additionally detail how this hypothesis test can be optimised in order to maximise its power, and describe how the second stage sample sizes can be chosen to more readily provide the operating characteristics that were originally envisioned by Gehan. Finally, we contrast our modified Gehan designs to Simon’s designs, based on two examples motivated by real clinical trials.

**Results:**

Gehan’s original designs are often greatly under- or over-powered when compared to type-II error-rates typically used in phase II. However, we demonstrate that the control parameters of his design can be chosen to resolve this problem. With this, though, the modified Gehan designs have operating characteristics similar to the more familiar Simon designs.

**Conclusions:**

The trial design settings in which Gehan’s design will be preferable over Simon’s designs are likely limited. Provided the second stage sample sizes are chosen carefully, however, one scenario of potential utility is when the trial’s primary goal is to ascertain the treatment response rate to a certain precision.

**Electronic supplementary material:**

The online version of this article (10.1186/s12874-019-0659-2) contains supplementary material, which is available to authorized users.

## Background

Phase II oncology clinical trials are commonly carried out via non-randomized single-arm designs. In particular, Gehan’s two-stage single-arm design was perhaps the first design ever forwarded for phase II oncology trials [1]. In it, stage one is conducted to ascertain whether the regimen under study displays enough anti-cancer activity to justify further investigation, with this decision based upon whether at least one tumour response is observed amongst a small number of patients. Following the observation of at least one response, stage two is then constructed to try and ensure that the true response rate can be estimated to a certain precision.

Whilst Gehan’s design was once commonly utilised [2], it was later replaced as the typical approach to phase II trial conduct by two two-stage group sequential designs offered by Simon [3]. Importantly, the parameters of Simon’s designs are those which, amongst the parameter combinations that control the operating characteristics of a particular hypothesis test, minimise the expected sample size under a nominated uninteresting response rate, or minimise the trial’s maximal possible sample size. The simplicity of Simon’s designs, and their efficiency at weeding out inactive agents, has led to their evident sustained popularity [4–6].

Moreover, the fact that Simon’s designs are still commonly utilised has meant that developing methodology for their extension remains an active area of research. Several recent such presentations have focused upon a so-called flexible two-stage design framework that allows, in particular, the second stage sample size to be dependent on the number of responses observed in stage one [7–11]. Interestingly, these flexible designs therefore have parallels with Gehan’s once popular design, which also specifies the stage-two sample sizes in a response adaptive manner.

Ultimately, Gehan’s design fell out of common use because, unlike Simon’s designs, it provides no means of formally testing whether a regimen’s observed response rate is sufficiently large to warrant its further development [2]. That is, it affords no method for controlling a study’s type-I error-rate or power to a desired level. Indeed, the latest available figures on phase II oncology trials suggest Gehan’s approach is now used infrequently in comparison to Simon’s designs. Specifically, Langrand-Escure et al. (2017) [6] reviewed phase II clinical trials published in three top oncology journals between 2010 and 2015. They identified only six studies that utilised Gehan’s design. However, on our further inspection, only three of these articles cited Gehan’s paper. Therefore, to more accurately quantify how often Gehan’s design has been employed in recent years, we carried out a narrative literature review, ultimately finding evidence that Gehan’s design is being used more regularly than previous reviews suggest.

Specifically, we surveyed the 200 articles, according to Google Scholar, which have cited Gehan’s 1961 paper since January 1 2008. Additionally, we reviewed the 1872 articles on PubMed Central, with a publication date later than January 1 2008, that contained “Gehan" in any field. We found 52 papers that stated they had utilised either Gehan’s methodology, or a modified version of it, with many in high impact oncology journals. Further details of how this survey was conducted are provided in Additional files 1 and 2. Moreover, two of the articles found by Langrand-Escure et al. (2017) [6] were not identified in our search. Consequently, it is possible that substantially more published trials have utilised Gehan’s design in recent years than our narrative review suggests. And, of course, there may well be numerous unpublished trials that have utilised his approach, given that many studies remain unpublished [12], and as it has been argued previously, single-arm trials may be more susceptible to non-publication than their randomised counterparts because their small sample size leads to a perception that they have less intrinsic value [13].

Therefore, methods that improve Gehan’s original design, and provide further evidence on its statistical characteristics, are of value to the trials community. Here, our focus is on providing such methodology. Significantly, we describe how techniques for flexible two-stage single-arm trials can be used to incorporate hypothesis testing in to Gehan’s design. We further expound on how this test can be optimised in order to maximise its power. Following this, we describe modified approaches to specifying the second-stage sample sizes in Gehan’s design, in order to permit the design’s desired operating characteristics to be more commonly attained.

The primary motivation for our work is then to utilise our results to be able to present a thorough comparison of our modified versions of Gehan’s design to Simon’s designs. We achieve this based on two real trial examples, and discuss important considerations around the power of the designs, along with the precision to which they can estimate the response rate on trial conclusion. We conclude with a discussion of the potential scenarios in which our enhanced versions of Gehan’s design could be useful within the context of developing a novel treatment regime.

## Methods

### Gehan’s design

We proceed by first formally describing Gehan’s design. As noted, Gehan proposed a two-stage approach in which a regimen’s performance is judged according to the number of patients who experience a tumour response. Thus, denoting the outcome for patient *i* by *X*_*i*_, Gehan’s framework supposes that *X*_*i*_∼*Bern*(*π*), for response rate *π*∈[0,1]. A response rate, *π*_1_∈(0,1], is specified so as to warrant the further investigation of the regimen. Then, the sample size required in stage one, $n_{1}\in \mathbb {N}^{+}$, is chosen based on $S_{1} = {\sum \nolimits }_{i=1}^{n_{1}}X_{i} \sim Bin(n_{1},\pi)$, using a rejection probability *β*_1_∈(0,1), as

1 $$ \underset{n_{1}\in\mathbb{N}^{+}}{\text{argmin}}\{ b(0 \mid n_{1}, \pi_{1}) \le \beta_{1}\},  $$

where *b*(*s*∣*m*,*π*)=^*m*^*C*_*s*_*π*^*s*^(1−*π*)^*m*−*s*^ is the probability mass function of a *Bin*(*m*,*π*) random variable. Thus, *n*_1_ is chosen such that if the response rate is at least *π*_1_, then the probability of observing no responses is less than or equal to *β*_1_.

Then, if the observed value of *S*_1_,*s*_1_, is equal to zero, the study is stopped for futility. Otherwise, Gehan suggested that the sample size for stage two, $n_{2}\in \mathbb {N}$, be chosen to allow the true response rate to be estimated to a certain precision. Explicitly, an interim estimate of the response rate, $\hat {\pi }\in [0,1]$, is specified based on the first stage data. We then choose *n*_2_ as

2 $$ \underset{n_{2}\in\mathbb{N}}{\text{argmin}} \left\{\sqrt{\frac{\hat{\pi}(1 - \hat{\pi})}{n_{1}+n_{2}}} \le \gamma\right\}.  $$

Here, $\sqrt {\hat {\pi }(1 - \hat {\pi })/(n_{1}+n_{2})}$ is an estimate of the standard error of the response rate at the end of stage two. Thus, Gehan proposed that this estimate be controlled to some maximal value *γ*∈(0,1]. Note that the above allows for *n*_2_=0, signifying that the desired precision is met at the end of stage one.

Observing that the above calculation is heavily dependent upon $\hat {\pi }$, Gehan advised that a conservative value be specified via the upper 75th percent confidence limit for *π*, based on the stage one data. He did not describe precisely how this confidence interval should be computed, but the designs that were subsequently presented suggest that a Wald-based confidence interval was utilised, giving for any *s*_1_ and *n*_1_

3 $$ \begin{aligned} \hat{\pi} \equiv \hat{\pi}(s_{1},n_{1}) = \min\left\{\frac{s_{1}}{n_{1}} + \Phi^{-1}(1 - 0.125)\sqrt{\frac{s_{1}(n_{1} - s_{1})}{n_{1}^{3}}},1\right\}. \end{aligned}  $$

This proposal remains a potentially reasonable one if our desire is to approximately provide a certain level of precision in the estimate of the response rate at the end of stage two. However, this specification of $\hat {\pi }$, based on an argument for conservatism, can be improved upon without a significant increase in computational or statistical complexity. Specifically, given the typically small nature of *n*_1_, a confidence limit based on a confidence interval determination procedure that is not reliant on asymptotic theory could be utilised. Moreover, Eq. (2) will be maximised, for any $n_{1}+n_{2}\in \mathbb {N}^{+}$, when $\hat {\pi }=0.5$. Therefore, using the upper confidence limit when *s*_1_/*n*_1_≥0.5 is actually less conservative than the simple maximum likelihood estimate *s*_1_/*n*_1_. Such a possibility was an unlikely one in the 1960s but may not be unreasonable in certain disease settings today. Consequently, choosing Clopper-Pearson [14] as the approach to confidence interval specification, these considerations could lead to the following proposal for $\hat {\pi }$, rather than that given in Eq. (3)

4 $$ \hat{\pi}(s_{1},n_{1}) = \left\{\begin{array}{ll} \underset{\hat{\pi}\in\hat{\Pi}}{\text{argmin}} |\hat{\pi} - 0.5| &: s_{1}\in\{1,\dots,n_{1}-1\},\\ 0.125^{1/n_{1}} &: s_{1}=n_{1}, \end{array}\right.\\  $$

for $\hat {\Pi } = \{Q_{\text {Beta}}(0.125, s_{1}, n_{1} - s + 1),Q_{\text {Beta}}(0.875, s_{1} + 1, n_{1} - s_{1}),s_{1}/n_{1}\}$. Here, *Q*_Beta_(*p,a*,*b*) is the *p*th quantile of a Beta distribution with shape parameters *a* and *b*. That is, $\hat {\pi }$ could be specified as either its maximum likelihood estimate *s*_1_/*n*_1_, or its lower or upper 75th percent confidence limits using Clopper-Pearson, according to which is closest to 0.5 (the elements in $\hat {\pi }$).

In this paper, we consider both of these methods for specifying $\hat {\pi }$. We refer to Gehan’s original approach based on Eq. (3) as the ‘original’, and our proposal in Eq. (4), as the ‘conservative’ method. Note that in the above we retain use of the 75% confidence interval. However, intervals for other coverages could readily be employed.

The above completes the description of our approach to specifying Gehan’s design. Notably, Gehan provided a table of designs for several combinations of *π*_1_,*β*_1_, and *γ*. We will return later to consider the power of these designs following the inclusion of a hypothesis test.

### Incorporating and optimising a hypothesis test

To resolve one of the principal limitations of Gehan’s design framework, we now describe how we can modify his approach to include the hypothesis test typically associated with phase II oncology trials. Precisely, we test the following null hypothesis

5 $$ H_{0} : \pi = \pi_{0},  $$

where *π*_0_∈(0,*π*_1_). As usual, we will desire to control the type-I error-rate under *H*_0_ to some *α*∈(0,1). Note that here, *π*_0_ is an uninteresting or null response rate that would make the regimen of no further interest. Typically, this is specified based on the historical response rate for the current standard of care.

Now, the methodology of the previous section allows us to prescribe values for *n*_1_, and *n*_2_ for each *s*_1_∈{0,…,*n*_1_}, which we will signify from here by *n*_2_(*s*_1_). Such notation is common in the flexible and adaptive two-stage single-arm trial literature [7–9], and indeed we can readily view Gehan’s design as a type of flexible two-stage design. For, whilst these articles have generally sought to determine values *n*_2_(*s*_1_) that minimise some function of the trial’s (expected) required sample size, as is evident, Gehan’s framework simply prescribes an alternative approach to specifying the second stage sample sizes based on the first stage data.

Importantly, the literature on flexibly designing two-stage single-arm trials is facilitated by the concept of a discrete conditional error function (DCEF), as formalised by Englert and Keiser (2012) [7]. A DCEF consists of values *D*(*s*_1_)∈[0,1] for *s*_1_∈{0,…,*n*_1_}. Using these values, if *D*(*s*_1_)=0 the trial is terminated at the end of the first stage for futility (*H*_0_ is not rejected). Similarly, if *D*(*s*_1_)=1 the trial is terminated at the end of stage one for efficacy (*H*_0_ is rejected). Otherwise, for those *s*_1_ such that *D*(*s*_1_)∈(0,1), the trial continues to the end of stage two, and rejects *H*_0_ if the second stage *p*-value, *p*_2_, is sufficiently small. Formally, *H*_0_ is rejected when

$$\begin{array}{*{20}l} p_{2}\{s_{2} | n_{2}(s_{1}),\pi_{0}\} &= \mathbb{P}\{S_{2} \ge s_{2} \mid n_{2}(s_{1}),\pi_{0}\},\\ &= 1 - B\{s_{2} - 1 \mid n_{2}(s_{1}),\pi_{0}\},\\ &\le D(s_{1}), \end{array} $$

where $S_{2} = {\sum \nolimits }_{i=n_{1}+1}^{n_{1}+n_{2}(s_{1})} X_{i} \sim Bin\{n_{2}(s_{1}), \pi \}$, and *B*(*s*∣*m*,*π*) is the cumulative distribution function of a *Bin*(*m*,*π*) variable. Then, the test is controlled to level *α* provided that

6 $$ \sum\limits_{s_{1}=0}^{n_{1}}D(s_{1})b(s_{1} \mid n_{1},\pi_{0}) \le \alpha.  $$

It is this concept of a DCEF that allows us to incorporate a hypothesis test in to Gehan’s design. Our task is simply to choose values for the *D*(*s*_1_) such that Eq. (6) holds: any such set of values, in combination with the testing rules described, allows us to include a formal test of the hypothesis given in Eq. (5), and be assured that the type-I error-rate is controlled to the desired level.

In practice, there will be many such sets of values that will conform to the above requirement, and therefore a method is necessitated for choosing between them. To achieve this in a logical manner, we can specify an optimality criteria of interest. As noted above, the previous articles in this domain have focused on methods for optimally choosing the *D*(*s*_1_) to minimise some function of the trial’s expected sample size. In fact, in Englert and Keiser (2013) [8] and Shan et al. (2016) [9], each *D*(*s*_1_) is directly associated with a value for *n*_2_(*s*_1_). That is, *n*_2_ is dependent on *s*_1_ through the value of *D*(*s*_1_). Thus, their optimisation procedures also determine the second stage sample sizes.

In our setting, Gehan’s precision requirement is instead responsible for the specification of the *n*_2_(*s*_1_). Consequently, we cannot use considerations around the expected sample size to optimise the *D*(*s*_1_). Therefore, we propose here to instead maximise the power of the resulting hypothesis test. To this end, note that the probability we reject *H*_0_ for any *π*∈[0,1] is given by

7 $$ P(\pi) = \sum\limits_{s_{1}=0}^{n_{1}}\mathbb{P}\{P_{2} \le D(s_{1}) \mid n_{2}(s_{1}),\pi\}b(s_{1} \mid n_{1},\pi),  $$

where *P*_2_ denotes the random value of the second stage *p*-value, the distribution of which is dependent upon *π* and *n*_2_(*s*_1_) [8]. Then, it is *P*(*π*_1_) that we use as our optimality criteria.

The final key consideration is to carefully specify the restrictions that are placed upon the *D*(*s*_1_). Here, the following are used

1. *D*(0)<*D*(1)<⋯<*D*(*n*_1_). This restriction is logical in that the probability we will reject *H*_0_ should increase as the number of responses observed at interim does.
2. *D*(*s*_1_)∈{0,1−*B*[*n*_2_(*s*_1_)−1∣,*n*_2_(*s*_1_),*π*_0_],…,1−*B*[0∣,*n*_2_(*s*_1_),*π*_0_],1}. This restriction corresponds to the fact that we need not treat the *D*(*s*_1_) as continuous parameters, as for each *s*_1_ there are a finite number of possible *p*-values that can be observed at the end of stage two; specifically those specified in the set here.
3. *D*(*s*_1_)∈{0,1} if *n*_2_(*s*_1_)=0. If *n*_2_(*s*_1_)=0 the trial is stopped at the end of stage one. To ensure that a decision is always made in our testing framework, we must therefore have that *H*_0_ is either rejected (*D*(*s*_1_)=1) or not rejected (*D*(*s*_1_)=0) at this point. A caveat of this restriction is that we must have *D*(0)=0, as *D*(0)=1 would imply a type-I error-rate of one given Restriction 1.
4. *D*(*s*_1_)∉{0,1} if *n*_2_(*s*_1_)>0. If *n*_2_(*s*_1_)>0 then the trial progresses to stage two. In this case, *D*(*s*_1_) should not equal 0 or 1 as it is not logical for a decision on the trial’s outcome to be certain before the second stage commences.

Thus, our problem is reduced to maximising Eq. (7) over an *n*_1_-dimensional discrete search space. Unfortunately, this will in general still leave an extremely large number of possible choices for the *D*(*s*_1_). Fortunately, Englert and Keiser (2013) [8] have demonstrated how this problem can be resolved using the branch-and-bound algorithm to efficiently and exhaustively search over the possible designs. Briefly, this algorithm works by recursively defining the *D*(*s*_1_) for *s*_1_∈{0,…,*n*_1_} through repeated branching steps that split the optimisation problem in to further and further sub-problems. Within this recursion, the bounding step systematically discards sub-problems that cannot lead to the optimal design. Here, this corresponds to those sub-problems which either cannot control the type-I error-rate to the desired level *α*, or cannot increase the trial’s power relative to that of the best design identified thus far. More precisely, after *s* branching steps, when *D*(*s*_1_) has been specified for *s*_1_∈{0,…,*s*}, the minimal possible type-I error-rate of a design for any potential choices of *D*(*s*_1_) for *s*_1_∈{*s*+1,…,*n*_1_}, is given by

$$ \alpha_{\text{min}} = \sum\limits_{s_{1}=0}^{s}D(s_{1})b(s_{1} \mid n_{1},\pi_{0}) + D(s)\sum\limits_{s_{1}=s+1}^{n_{1}}b(s_{1} \mid n_{1},\pi_{0}), $$

and the maximal possible power will be

$$\begin{array}{*{20}l} P_{\text{max}} =& \sum\limits_{s_{1}=0}^{s}\mathbb{P}\{P_{2} \le D(s_{1}) \mid n_{2}(s_{1}),\pi_{1}\}b(s_{1} \mid n_{1},\pi_{1})\\ &+ \sum\limits_{s_{1}=s+1}^{n_{1}}b(s_{1} \mid n_{1},\pi_{1}). \end{array} $$

We can therefore discard all sub-problems when *α*_min_>*α* or *P*_max_<*P*_current_, where *P*_current_ is the largest power of the designs considered so far. It is this bounding step that allows for the efficient consideration of all possible designs, as we are able to avoid the computational cost of evaluating many sets of *D*(*s*_1_) that could not possibly be optimal.

Note that one small caveat to the above considerations is that a design may not exist that is capable of controlling the type-I error-rate to *α*. Explicitly, the most conservative possible design would take for *s*_1_∈{1,…,*n*_1_}

$$\begin{aligned}D(s_{1}) = \left\{ \begin{array}{ll} 0 &: {\sum\nolimits}_{s=1}^{s_{1}}n_{2}(s_{1})=0,\\ b[n_{2}(s_{1}) \mid n_{2}(s_{1}),\pi_{0}] &: n_{2}(s_{1})>0,\\ 1&: n_{2}(s_{1})=0 \text{ and} {\sum\nolimits}_{s=1}^{s_{1}-1}n_{2}(s_{1})>0. \end{array}\right. \end{aligned} $$

Thus the minimal possible type-I error-rate is *P*(*π*_0_) with the above values of the *D*(*s*_1_), and therefore if this is greater than *α* no DCEF exists which attains the desired type-I error-rate. However, later, we perform a large search over what are likely to be common choices for *α*,*γ*,*π*_0_, and *π*_1_, and demonstrate that this is likely to rarely occur in practice, at least when using the conservative approach to specifying $\hat {\pi }$ in *f*_*G*_.

This describes our complete approach to optimising a test of the hypotheses given in Eq. (5) within Gehan’s design. A program to execute our search procedure in R is available in the singlearm package [15].

### Alternative methods for specifying the second stage sample sizes

Later, we will observe that Gehan’s design determination procedure, even with our conservative method for specifying $\hat {\pi }$ at the end of stage one, would routinely be expected not to provide the desired level of precision in the estimate of the response rate at the end of stage two. For this reason, we here detail several alternative methods that could be used to specify the second stage sample sizes.

First, suppose that *n*_1_ is specified as the solution of Eq. (1). Then, a general framework for specifying *n*_2_, for any *s*_1_, can be prescribed by allocating it as the solution of the following problem

$$\underset{n_{2}\in\mathbb{N}}{\text{argmin}} \{f(n_{2} \mid \boldsymbol{\theta}) \le \gamma\}. $$

Here, *f* is a function that evaluates the suitability of a candidate *n*_2_, for a given vector of (decision guiding) parameters ***θ***. In Gehan’s original proposal

$$f = f_{G}\left\{n_{2} \mid (\hat{\pi},n_{1})^{\top}\right\} = \sqrt{\frac{\hat{\pi}(1 - \hat{\pi})}{n_{1}+n_{2}}}. $$

It is a consequence of that fact that *f*_*G*_ provides only an estimate of the true standard error that the desired precision may not be achieved at the end of the trial. One way to resolve this issue would be to specify *f* via a function *L*(*s*_1_,*s*_2_,*n*_1_,*n*_2_), which prescribes the length of the confidence interval for *π* at the end of the trial, given the number of responses observed in stages one and two. Then, *n*_2_ could be determined using

$$\begin{aligned} f=f_{L}\left\{n_{2} \mid (s_{1},n_{1})^{\top}\right\} = \frac{0.5}{\Phi^{-1}(1 - \alpha/2)}\max_{s_{2}\in\{0,\dots,n_{2}\}} L(s_{1},s_{2},n_{1},n_{2}). \end{aligned} $$

That is, *n*_2_ could be chosen to ensure that, no matter the value of *s*_2_, half of the confidence interval width is always constrained to *Φ*^−1^(1−*α*/2)*γ*. The factor *Φ*^−1^(1−*α*/2) arises here to correspond to Gehan’s original precision requirement, which aims to ensure a Wald confidence interval for *π* at the end of stage two has length 2*Φ*^−1^(1−*α*/2)*γ* (i.e., so that the designs aim to achieve the same precision requirement).

In practice, such an approach may lead in certain circumstances to undesirably large values of the *n*_2_(*s*_1_). An intermediate option might be to make use of an interim estimate of *π*, as well as a function *L*(*s*_1_,*s*_2_,*n*_1_,*n*_2_). Then, half the expected length of the final confidence interval could be constrained to *γ*, when the true response rate is $\hat {\pi }$, by taking

$$\begin{array}{*{20}l} f &= f_{EL}\left\{n_{2} \mid (\hat{\pi},s_{1},n_{1})^{\top}\right\}\\ &= \frac{0.5}{\Phi^{-1}(1 - \alpha/2)}\sum\limits_{s_{2}=0}^{n_{2}}b(s_{2} \mid n_{2},\hat{\pi})L(s_{1},s_{2},n_{1},n_{2}). \end{array} $$

In this paper, we will consider the operating characteristics of designs determined using *f*_*G*_,*f*_*L*_, and *f*_*EL*_ for the specification of the second stage sample sizes, considering the utility of both Eqs. (3) and (4) for the value of $\hat {\pi }$ in *f*_*EL*_. Furthermore, we utilise Clopper-Pearson for *L*(*s*_1_,*s*_2_,*n*_1_,*n*_2_) in the above equations, giving

8 $$\begin{array}{*{20}l} {} L(s_{1},\!s_{2},\!n_{1},\!n_{2})\! &\equiv L(s\equiv s_{1}+s_{2},n\equiv n_{1}+n_{2}),\\ &= \left\{\! \begin{array}{ll} Q_{\text{Beta}}(1-\alpha/2, s + 1, n - s)\\ - Q_{\text{Beta}}(\alpha/2, s, n - s + 1) &: s \notin \{0,n\},\\ 1 - (\alpha/2)^{1/n} &: \text{otherwise}. \end{array}\right. \end{array} $$

### Design comparison

In what follows, we assess the power of Gehan’s original designs for the majority of parameters considered in Table II of his paper. We motivate a more in depth examination of the performance of our modified and optimised designs using design parameters based on two real clinical trials.

Firstly, Dupuis-Girod et al. (2012) [16] presented the results of a phase II study to test the efficacy of bevacizumab in reducing high cardiac output in severe hepatic forms of hereditary hemorrhagic telangiectasia. Gehan’s design was employed, with *β*_1_=0.1,*π*_1_=0.3, and *γ*=0.1. We will consider designs for *α*=0.05, when *π*_0_=*π*_1_−0.15=0.15.

In Additional file 1 we also present results corresponding to Lorenzen et al. (2008) [17], who investigated the tumour response rate to neoadjuvant continuous infusion of weekly 5-fluorouracil and escalating doses of oxaliplatin plus concurrent radiation in patients with locally advanced oesophageal squamous cell carcinoma. This trial also used Gehan’s design, but for *β*_1_=0.05,*π*_1_=0.5, and *γ*=0.1. In this case, we consider designs for *α*=0.1, with *π*_0_=*π*_1_−0.2=0.3.

In both cases, we denote the Simon designs as having stage-wise group sizes *n*_1_ and *n*_2_, and futility boundaries *f*_1_ and *f*_2_ (that is, stage two is commended if *s*_1_>*f*_1_, and *H*_0_ rejected only when *s*_1_+*s*_2_>*f*_2_). Then, for these designs, we have

$$n_{2}(s_{1}) = \left\{\begin{array}{ll} 0 &: s_{1} \le f_{1},\\ n_{2} &: s_{1} > f_{1}. \end{array}\right. $$

In our assessments, we repeatedly examine several different statistical quantities in order to compare the performance of the designs. In all instances, we calculate these quantities using exact calculations, without recourse to simulation, by employing exhaustive calculations over possible trial outcomes.

Firstly, we will examine the expected sample size (ESS) required by the various designs. Therefore, note that we can compute this for any *π*∈[0,1] using

$$ESS(\pi) = \sum\limits_{s_{1}=0}^{n_{1}}\{n_{1}+n_{2}(s_{1})\}b(s_{1} \mid n, \pi). $$

We also compare the expected length of the 100(1−*α*)% confidence intervals at the end of the trials, conditional on not stopping for futility in stage one. That is, conditional on *S*_1_>*f*_1_, where for the Gehan designs we take $f_{1}=\text{argmax}_{s_{1}\in \{0,\dots,n_{1}\}}\{D(s_{1})=0\}$. We compute this, for any *π*∈[0,1], as

$$\begin{aligned} & EL(\pi \mid S_{1}>f_{1})\\ &= \frac{{\sum\nolimits}_{s_{1} = f_{1}}^{n_{1}}{\sum\nolimits}_{s_{2} = 0}^{n_{2}(s_{1})} L\{s_{1} + s_{2}, n_{1} + n_{2}(s_{1})\}b(s_{1} \mid n_{1}, \pi)b\{s_{2} \mid n_{2}(s_{1}), \pi\}}{{\sum\nolimits}_{s_{1} = 1}^{n_{1}} b(s_{1} \mid n_{1}, \pi)}. \end{aligned} $$

We will refer to this as the conditional expected length (CEL). We focus on the CEL, rather than the unconditional expected length of the confidence interval across all possible values of *s*_1_, for two reasons. Firstly, because Gehan’s designs is constructed to try and provide a certain precision at the end of stage two. And secondly, as analysis of this kind is arguably more important when a trial has not been stopped early for futility [18].

Adaptive two-stage designs require specialised methodology for confidence interval construction, and therefore when computing the CEL, we utilise for *L*(*s*_1_,*s*_2_,*n*_1_,*n*_2_) the exact Clopper-Pearson type confidence interval, based on an ordering of the sample space induced by the optimal compatible estimator, described by Kunzmann and Keiser (2018) [11]. Our reason for utilising such confidence intervals for computing the CEL, but not when evaluating *f*_*L*_ and *f*_*EL*_, is as follows: the adjusted confidence intervals of Kunzmann and Keiser (2018) [11] are only defined given the *n*_2_(*s*_1_). Thus after accounting for the complexity of their calculation, this means that they cannot be used in a computationally efficient to choose the *n*_2_(*s*_1_).

Furthermore, note that by the above we are utilising the same type of confidence interval construction procedure for both the Gehan and Simon designs, in order to make our comparisons fair. Finally, unfortunately no closed form expressions are available for such *L*. However, they can be computed using available software [11]. We have stored all our required confidence intervals in.csv files contained within Additional file 5, and provided the Julia code for their determination in Additional file 4.

When comparing the various Gehan designs to each other, we will also consider *EL*(*π*∣*S*_1_=*s*_1_), the conditional expected confidence interval lengths for each possible value of *s*_1_>0, given by

$$EL(\pi\! \mid\! S_{1}\,=\,s_{1})\! =\! \sum\limits_{s_{2} = 0}^{n_{2}(s_{1})} L\{s_{1} + s_{2}, n_{1} + n_{2}(s_{1})\}b\{s_{2} \mid n_{2}(s_{1}), \pi\}. $$

Note that code to re-create our design evaluations and reproduce each of the tables and figures is provided in Additional file 3.

## Results

### Power of Gehan’s design

First, we present the optimal values of the *D*(*s*_1_), along with the corresponding type-I error-rate, power, and values of *ESS*(*π*_0_) and *ESS*(*π*_1_), for several of the parameter combinations given in Table II of Gehan (1961) [1]. Explicitly, these correspond to (*β*_1_,*γ*,*π*_1_)∈{0.05,0.1}×{0.05,0.1}×{0.2,0.25,0.3} with *α*=0.05. Our results are provided in Table 1 for both the original and conservative methods for specifying $\hat {\pi }$ at the end of stage one, in Gehan’s original *f*_*G*_ for specifying the second stage sample sizes. In Additional file 1, we present further results for many other possible parameter combinations.

**Table 1:** Optimal hypothesis tests in Gehan designs using *f*_*G*_

*β*_1_	*γ*	*π*_1_	Method	*P*(*π*_0_)	*P*(*π*_1_)	*ESS*(*π*_0_)	*ESS*(*π*_1_)	*D*(1)	…	*D*(7)	*D*(8)	*D*(9)	*D*(10)	*D*(11)	*D*(12)	*D*(13)	*D*(14)
0.050	0.050	0.200	Original	0.011	0.918	37.595	79.781	0.011	…	0.341	0.433	0.498	0.558	0.623	1	1	1
0.050	0.050	0.200	Conservative	0.038	0.952	46.420	87.837	0.069	…	0.260	0.411	0.431	0.431	0.591	0.722	0.793	0.825
0.100	0.050	0.200	Original	0.006	0.884	35.709	78.604	0.013	…	0.125	0.201	0.337	1	1			
0.100	0.050	0.200	Conservative	0.049	0.913	43.429	86.933	0.068	…	0.638	0.656	0.804	0.881	0.910			
0.050	0.100	0.200	Original	0.790	0.148	27.484	22.238	0	…	0.008	0.044	0.143	1	1	1	1	1
0.050	0.100	0.200	Conservative	0.0001	0.093	16.726	22.438	0.00001	…	0.002	0.002	0.002	0.002	0.012	0.057	0.185	1
0.100	0.100	0.200	Original	0.001	0.188	13.813	20.689	0.001	…	0.226	1	1	1	1			
0.100	0.100	0.200	Conservative	0.001	0.219	15.633	22.557	0.001	…	0.004	0.004	0.025	0.086	0.185			
0.050	0.050	0.250	Original	0.049	0.937	54.563	85.193	0.013	…	0.125	0.201	0.337	1	1			
0.050	0.050	0.250	Conservative	0.050	0.948	64.667	92.219	0.068	…	0.638	0.656	0.804	0.881	0.910			
0.100	0.050	0.250	Original	0.049	0.909	52.961	83.088	0.037	…	0.537	1	1					
0.100	0.050	0.250	Conservative	0.050	0.918	61.160	90.499	0.116	…	0.839	0.921	0.946					
0.050	0.100	0.250	Original	0.011	0.376	16.512	21.979	0.001	…	0.226	1	1	1	1			
0.050	0.100	0.250	Conservative	0.016	0.403	18.859	23.531	0.001	…	0.004	0.004	0.025	0.086	0.185			
0.100	0.100	0.250	Original	0.035	0.517	15.904	21.446	0.001	…	1	1	1					
0.100	0.100	0.250	Conservative	0.040	0.607	18.026	23.348	0.030	…	0.043	0.153	0.337					
0.050	0.050	0.300	Original	0.050	0.926	66.975	86.330	0.064	…	0.277	0.376	0.570	1	1			
0.050	0.050	0.300	Conservative	0.050	0.938	75.364	94.102	0.053	…	0.878	0.950	0.975	0.979	0.993			
0.100	0.050	0.300	Original	0.049	0.890	62.925	81.433	0.047	…	0.794	1	1					
0.100	0.050	0.300	Conservative	0.050	0.901	68.827	90.501	0.063	…	0.984	0.991	0.998					
0.050	0.100	0.300	Original	0.040	0.524	18.393	22.065	0.009	…	0.410	1	1	1	1			
0.050	0.100	0.300	Conservative	0.048	0.571	20.558	24.042	0.013	…	0.044	0.044	0.134	0.264	0.344			
0.100	0.100	0.300	Original	0.021	0.404	17.416	20.829	0.053	…	1	1	1					
0.100	0.100	0.300	Conservative	0.049	0.572	19.230	23.517	0.044	…	0.211	0.415	0.570					

A summary of the optimal choices of the *D*(*s*_1_), along with the associated type-I error-rate, *P*(*π*_0_), and power, *P*(*π*_1_), are shown for a range of values of *β*_1_,*γ*, and *π*_1_. In all cases, *π*_0_=*π*_1_−0.15. Note that *D*(0) is not listed as it is zero in all instances. All values *D*(*s*_1_)∈(0,1) are given to 4 decimal places

From Table 1, we observe that in all instances our search procedure returns values for the *D*(*s*_1_) that imply a type-I error-rate of less than *α*=0.05. Moreover, the corresponding power of the designs ranges between 0.073 and 0.948. Thus, as was noted earlier, in no instance is the optimization procedure unable to find a design confirming to the desired level of type-I error control. However, there are instances in which the discrete nature of the test only permits a design with *P*(*π*_0_)≪*α*, which in turn results in some small values of *P*(*π*_1_). Nonetheless, it is clear that the power of Gehan’s designs is heavily dependent upon the choice of the design parameters.

In addition, note that the power of the design when using the conservative method for specifying $\hat {\pi }$ is always larger than that for the original method. This is a consequence of the fact that the conservative method, as was discussed, results in larger values for the *n*_2_(*s*_1_). This is evidently at a cost to the trials ESS under *π*_0_ and *π*_1_, however.

### Comparison to Simon’s designs

We now focus on design for our motivating scenario based on Dupuis-Girod et al. (2012) [16]. In this case, our optimal version of Gehan’s design using the original method for constructing $\hat {\pi }$, for use with *f*_*G*_, has *n*_1_=7 and

$$\begin{aligned} D(0) &= 0,\ D(1) = 0.0115,\ D(2) = 0.0419,\ D(3) = 0.0791,\\ \ D(4) &= 0.1798,\ D(5) = 0.2775,\ D(6) = D(7) = 1,\\ n_{2}(0) &= 0,\ n_{2}(1) = 14,\ n_{2}(2) = 18,\\ n_{2}(3) &= 16,\ n_{2}(4) = 10,\ n_{2}(5) = 2,\\ n_{2}(6) &= n_{2}(7) = 0,\\ P(\pi_{0}) &= 0.021,\ P(\pi_{1}) = 0.404,\\ ESS(\pi_{0}) &= 17.42,\ ESS(\pi_{1}) = 20.83. \end{aligned} $$

Similarly, using *f*_*G*_ with our conservative method for constructing $\hat {\pi }$

$$\begin{aligned} D(0) &= 0,\ D(1) = D(2) = 0.0419,\ D(3) = 0.2798,\ D(4) = 0.5203,\\ D(5) &= D(6) = 0.7759,\ D(7) = 0.8791,\\ n_{2}(0) &= 0,\ n_{2}(1) = n_{2}(2) = n_{2}(3) = n_{2}(4) = n_{2}(5) = n_{2}(6) = 18,\\ n_{2}(7) &= 13,\\ P(\pi_{0}) &= 0.049,\ P(\pi_{1}) = 0.572,\\ ESS(\pi_{0}) &= 19.23,\ ESS(\pi_{1}) = 23.52. \end{aligned} $$

Thus, the power of these modified Gehan designs is less than that we would generally desire in a phase II trial. Whilst for the former design this is in part due to the conservativeness of the test, even the conservative approach for constructing $\hat {\pi }$, which has larger second stage sample sizes, and attains a type-I error-rate close to the desired level, still only has power of 0.572. It is thus clear that neither method is capable of providing a reasonable amount of power for *π*_0_=*π*_1_−0.15. It is therefore useful to describe how this can be achieved, and also informative to examine the performance of the designs when they have a more typical level of power.

Explicitly, to achieve this for either method, we can treat *γ* as a parameter and identify a *γ*∈(0,1) that provides, say, 80% power. It is important to realise that such a search must be conducted carefully, as the discrete nature of the design means *P*(*π*_1_) may not be monotonic in *γ*. A simple option is to search for the maximal *γ* such that *P*(*π*_1_) is above the desired level. This is logical because the ESS will monotonically decrease in *γ*, as increasing *γ* has no effect on the design other than to monotonically decrease the *n*_2_(*s*_1_).

Performing this search for the original method, we find that *γ*=0.0658 gives a design with *n*_1_=7 and

$$\begin{aligned} D(0) &= 0,\ D(1) = 0.0418,\ D(2) = 0.0714,\ D(3) = 0.1421,\\ D(4) &= 0.7279,\ D(5) = 0.8578,\ D(6) = D(7) = 1,\\ n_{2}(0) &= 0,\ n_{2}(1) = 42,\ n_{2}(2) = 51,\\ n_{2}(3) &= 46,\ n_{2}(4) = 32,\ n_{2}(5) = 12,\\ n_{2}(6) &= n_{2}(7) = 0,\\ P(\pi_{0}) &= 0.049,\ P(\pi_{1}) = 0.800,\\ ESS(\pi_{0}) &= 37.52,\ ESS(\pi_{1}) = 47.43. \end{aligned} $$

Whilst for the conservative approach, *γ*=0.0686 results in a design with *n*_1_=7 and

$$\begin{aligned} D(0) &= 0,\ D(1) = 0.0354,\ D(2) = 0.0745,\ D(3) = 0.2457,\\ D(4) &= 0.3848,\ D(5) = 0.7067,\ D(6) = 0.9948,\ D(7) = 0.9960,\\ n_{2}(0) &= 0,\ n_{2}(1) = n_{2}(2) = n_{2}(3) = n_{2}(4) = n_{2}(5) = n_{2}(6) = 46,\\ n_{2}(7) &= 34,\\ P(\pi_{0}) &= 0.050,\ P(\pi_{1}) = 0.804,\\ ESS(\pi_{0}) &= 38.04,\ ESS(\pi_{1}) = 48.9. \end{aligned} $$

It is now highly informative to ask whether these optimised Gehan designs offer advantageous performance over Simon’s popular designs. Thus, next, we contrast the performance of these designs to the null-optimal and minimax Simon design’s when *β*=0.2. Precisely, these are

$$\begin{array}{*{20}l} \text{Null-optimal}&:\ f_{1} = 3,\ n_{1} = 19,\ f_{2} = 12,\ n_{2} = 36,\\ \text{Minimax} &:\ f_{1} = 3,\ n_{1} = 23,\ f_{2} = 11,\ n_{2} = 25. \end{array} $$

**Fig. 1:**
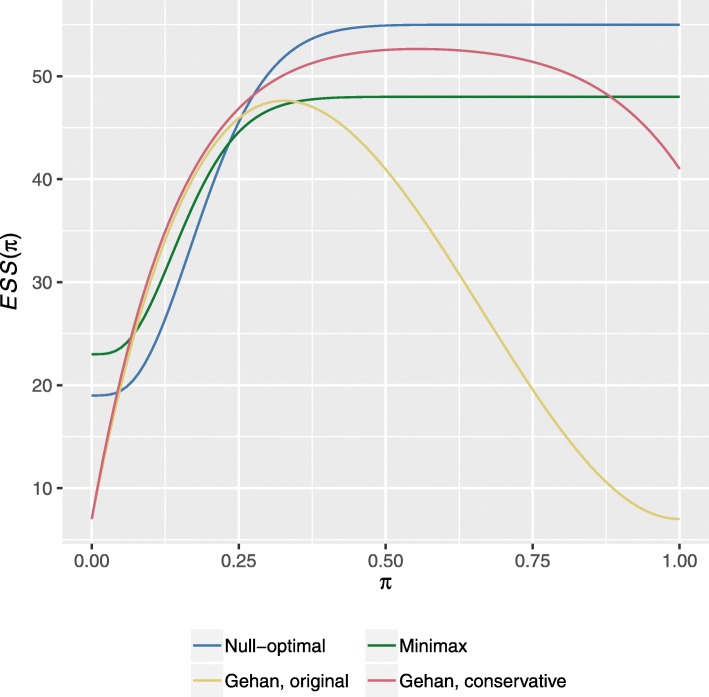
Expected sample size curves. Shows the *ESS*(*π*) curves for Gehan’s designs using Methods A and B, and Simon’s null-optimal and minimax designs

Thus the maximal sample size of both of the Gehan designs listed above is larger than that for both Simon designs. We further investigate the likely required sample size of these four designs through their ESS curves, which are provided in Fig. 1 for *π*∈[0,1]. We can see that the ESS of the Gehan designs is lower when *π* is close to zero; a result of their smaller first stage sample size. Similarly, the ability of the Gehan designs to lower their second stage sample size when *s*_1_ is large means that they return to having lower ESSs when *π* is large; this is particularly true for the design utilising the original approach to specifying $\hat {\pi }$. However, for a large range of arguably more realistic values of *π*, given the values of *π*_0_ and *π*_1_, the ESS of the Simon designs is smaller.

**Fig. 2:**
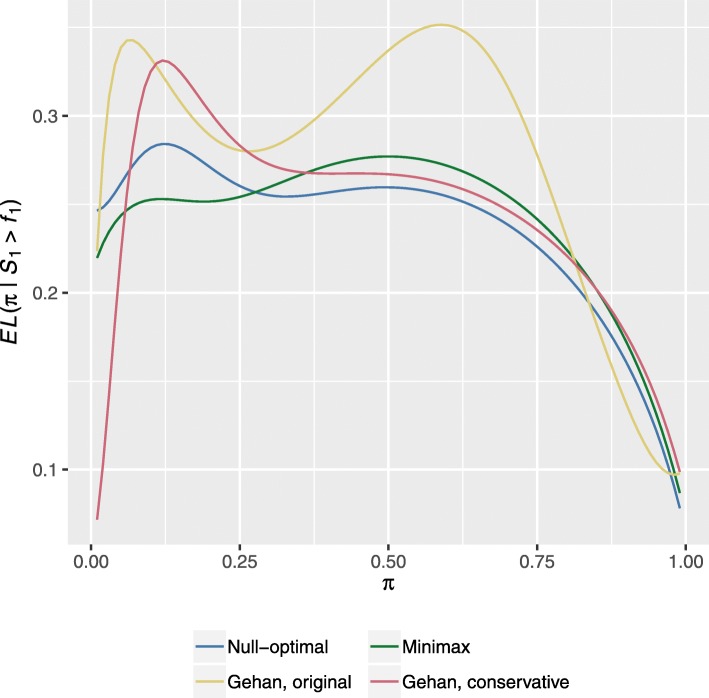
Expected confidence interval length curves, conditional on not stopping for futility at the end of stage one. Shows the *EL*(*π*∣*S*_1_>*f*_1_) curves for Gehan’s designs using Methods A and B (with *f*_*G*_), and Simon’s null-optimal and minimax designs

A final important question is whether the Gehan designs more readily estimate *π* to a certain precision, in contrast to that afforded by Simon’s designs. To this end, in Fig. 2 we compare the CEL curves of the four designs. We consider only *π*∈(0,1), as *π*∈{0,1} can result in strange results as the outcome of the designs is deterministic.

What we observe largely corresponds, as one would expect, to the findings in Fig. 2. That is, for the majority of values of *π* the design which has the largest ESS, has the smallest CEL value. In particular, for Gehan’s design with the original approach to specifying $\hat {\pi }$, when *π* is large, the ESS of this design being much smaller results in its CEL being substantially larger. Overall, it is clear that Simon’s designs, and the Gehan design with the conservative approach, have similar values for the CEL across a wide range of response rates.

### Gehan designs with modified second stage sample sizes

A further consequence of Fig. 2 is that the confidence intervals determined at the end of the Gehan designs evidently must in certain cases have length substantially greater than the implicitly desired 2*Φ*^−1^(1−*α*/2)*γ* based on Wald confidence intervals (which is, e.g., equal to 0.26 to 2 dp for the design using Gehan’s original approach to specifying $\hat {\pi }$).

We now conclude our results by investigating this further for the originally desired precision in the Dupuis-Girod et al. (2012) trial, *γ*=0.1. Firstly, we determined the optimised Gehan design based on *f*_*L*_ to be

$$\begin{aligned} D(0) &= 0,\ D(1) = 0.0287,\ D(2) = 0.0827,\ D(3) = 0.1975,\\ D(4) &= 0.6295,\ D(5) = 0.9671,\ D(6) = 0.9671,\ D(7) = 0.9671,\\ n_{2}(0) &= 0,\\ n_{2}(1) &= n_{2}(2) = n_{2}(3) = n_{2}(4) = n_{2}(5) = n_{2}(6) = n_{2}(7) = 21,\\ P(\pi_{0}) &= 0.049,\ P(\pi_{1}) = 0.619,\\ ESS(\pi_{0}) &= 21.27,\ ESS(\pi_{1}) = 26.27. \end{aligned} $$

In addition, that based on *f*_*EL*_ with the original approach to specifying $\hat {\pi }$ was identified as

$$\begin{aligned} D(0) &= 0,\ D(1) = 0.0003,\ D(2) = 0.0013,\ D(3) = 0.0041,\\ D(4) &= 0.0056,\ D(5) = 0.0099,\ D(6) = 0.0266,\ D(7) = 0.1500,\\ n_{2}(0) &= 0,\ n_{2}(1) = 14,\ n_{2}(2) = 20,\\ n_{2}(3) &= 19,\ n_{2}(4) = 16,\ n_{2}(5) = 10,\\ n_{2}(6) &= 5,\ n_{2}(7) = 1,\\ P(\pi_{0}) &= 0.0007,\ P(\pi_{1}) = 0.107,\\ ESS(\pi_{0}) &= 18.09,\ ESS(\pi_{1}) = 22.95. \end{aligned} $$

And finally, that for *f*_*EL*_ with our conservative approach to specifying $\hat {\pi }$ as

$$\begin{aligned} D(0) &= 0,\ D(1) = 0.0419,\ D(2) = 0.0673,\ D(3) = 0.1702,\\ D(4) &= D(5) = 0.3523, D(6) = 0.5203,\ D(7) = 0.6229,\\ n_{2}(0) &= 0,\ n_{2}(1) = 18,\ n_{2}(2) = n_{2}(3) = n_{2}(4) = n_{2}(5) = 20,\\ n_{2}(6) &= 18,\ n_{2}(7) = 6,\\ P(\pi_{0}) &= 0.046,\ P(\pi_{1}) = 0.586,\\ ESS(\pi_{0}) &= 19.80,\ ESS(\pi_{1}) = 24.85. \end{aligned} $$

As we would expect, as the most conservative approach, the required second stage sample sizes are largest for *f*_*L*_. Observe that for the conservative approach, relative to *f*_*G*_, using *f*_*EL*_ increases the stage two sample sizes for most *s*_1_, but decreases it for *s*_1_=7.

**Fig. 3:**
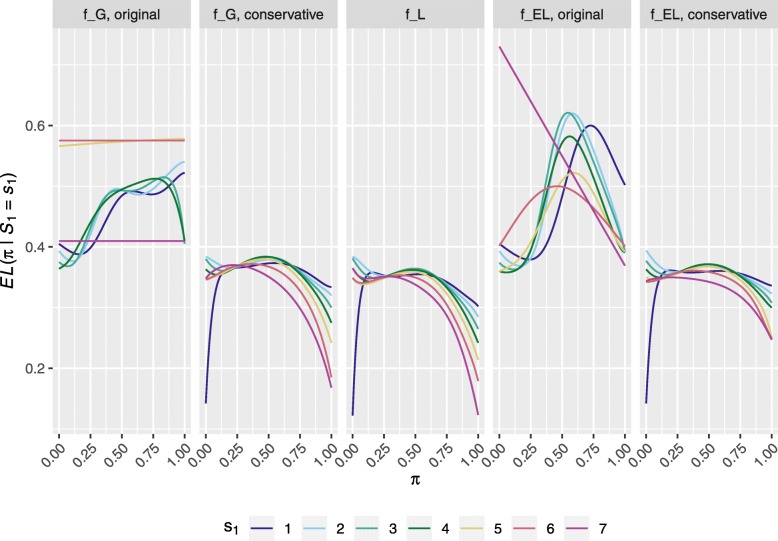
Expected confidence interval length curves, conditional on the number of responses in stage one. Shows the *EL*(*π*∣*S*_1_=*s*_1_) curves, for the five different Gehan designs based on the possible combinations of method (A or B) and function (*f*_*G*_,*f*_*L*_,*f*_*EL*_), when *s*_1_∈{1,…,*n*_1_}

We then present the CEL curves of the final 95% exact Clopper-Pearson type confidence intervals for the five designs (based on the considered combinations of function *f* with the original and conservative methods), for *s*_1_∈{1,…,*n*_1_}, in Fig. 3.

Gehan’s original design aimed to provide a (Wald) confidence interval with approximate length of 2*γΦ*^−1^(1−*α*/2)=0.39 to 2 dp. It is evident that Gehan’s original design (*f*_*G*_, Original) would often be expected to provide Clopper-Pearson type confidence intervals of length much larger than that desired. Moreover, we can see that utilising *f*_*EL*_ rather than *f*_*G*_ with the conservative approach improves performance for several values, but not all, of the *s*_1_.

Finally, using *f*_*L*_ guarantees that the final confidence interval has a CEL below that desired for all *s*_1_. So to do *f*_*G*_ and *f*_*EL*_ when paired with the conservative approach to specifying $\hat {\pi }$. In this case, where these designs require only a small increase to the second stage sample sizes (one that is arguably achievable given the maximal possible required sample size of Gehan’s original design), they should almost certainly be preferred.

## Discussion

Gehan’s design was once regularly used in phase II oncology trials. It did not, however, include a formal test of a regimen’s efficacy. Consequently, as the number of effective anti-cancer agents began to increase, and a higher standard of evidence was necessitated for a treatment to proceed to further testing, it fell out of habitual employment. Nonetheless, as was discussed, Gehan’s design is still utilised in practice. Thus methodology to improve upon Gehan’s original framework, and to describe the potential advantages of the modified approach compared to more commonly utilised designs, is therefore of value to the trials community. Here, we provided such work, describing the first methodology by which the hypothesis test typically associated with single-arm phase II trials can be incorporated in to Gehan’s design. We further went on to describe how this test can be optimised in order to maximise its power, and then presented a statistical evaluation of our modified Gehan designs.

It is valuable to note how our research builds upon previous findings. Several studies have identified that a major problem with Gehan’s design is that the probability stage two is commenced is typically high [19, 20], with this true even when the response rate is below that which we hope to observe. Here, we have provided the additional result that the power of Gehan’s originally presented designs varies widely for a null response rate of *π*_0_=*π*_1_−0.15 (Table 1). This suggests that many studies that have used Gehan’s design may have not had a strong probability to reliably identify efficacious treatments. In contrast, when the required precision *γ* was set to 0.05, some of the designs had power far higher than that which would typically be desired in a phase II trial.

We noted earlier that several of the designs in Table 1 have type-I error-rates substantially smaller than the permitted level. This is a consequence of the discrete nature of the design. In Additional file 1, via a large search over potential design parameters, we provide evidence that it is unlikely a reliable rule for when this will occur can be described. However, we argue that it would be expected to occur more often for larger values of *γ* and *π*_1_, when the second stage sample sizes are small. For, in this case, the number of permissible DCEFs will also be small, and the possibility that one will utilise the entire allowed type-I error will be reduced. A possible solution to this problem might be to relax the monotonicity requirements on the DCEF. However, as noted, this should in general be avoided. An ad hoc, but more acceptable solution, might be to artificially increase the values of the *n*_2_(*s*_1_) beyond those required by the precision requirements. This will increase the number of potential DCEFs, potentially permitting one which will more exhaustively utilise the allowed type-I error.

The fact that the power of Gehan’s original designs is not well calibrated may not be surprising, as it was not constructed to provide a certain power, but to estimate a response rate to within a certain precision. What is particularly troubling therefore is our presentations in Figs. 2 and 3, which demonstrated that typically the confidence interval width at the end of stage two would not be that which was desired. It is for this reason that we described how one can calculate the stage two sample sizes in an alternative manner to allow for more precise estimation at the end of the trial.

For our motivating example presented in this article, and that discussed in Additional file 1, we again identified potential issues with the power of Gehan’s designs for the utilised value of *γ*. For this reason, we advised that choosing *γ* carefully is particularly important, and described how a numerical search could be performed to identify the value of *γ* that provides the desired power.

The problem with this, however, is that once we modified the Gehan designs to have 80% power, on contrasting their performance to Simon’s designs, it was clear that Gehan’s designs often offered little advantage in terms of their statistical operating characteristics. Gehan’s designs tended to require fewer patients on average for extreme values of the response rate, but for arguably more realistic interim values of *π*, Simon’s designs were often more efficient (Fig. 1 and Additional file 1: Figure A5). Additionally, in Fig. 2 we observed few possible values of *π* for which the CEL of the Gehan designs was smaller than Simon’s designs. Though contrastingly, for the second scenario, in Additional file 1: Figure A6 it can be seen that Gehan’s designs would be expected to more accurately estimate the response rate at the end of stage tow.

The evident similar performance of the designs should perhaps not surprise us, as for the same type-I and type-II error-rates, the Gehan design’s parameters are similar to those of a non-optimal version of a two-stage group sequential design. This suggests that, for particular required error-rates, Gehan’s framework may have little utility for estimating the response rate *π* efficiently.

This begs the important question as to when Gehan’s designs could be useful, particularly when we take in to consideration the grater volume of theoretical results and software that is available pertaining to Simon’s designs. Firstly, in rare disease settings the fact that Simon’s designs may often have smaller ESSs makes them advantageous over Gehan’s design. It may in particular be anticipated that Gehan’s design would be useful when there are few available efficacious therapies for the disease under study, and thus any observed level of response would signify interest in proceeding to stage two. That is to say, when the value of *π*_0_ is small. For, this was in part Gehan’s motivation for the construction of his design. However, in this case, we could choose a non-optimal group sequential design with a small value of *f*_1_. We elaborate on this in Additional file 1. Consequently, we feel it is unlikely that Gehan’s design would regularly be preferable in such a setting.

Note that in order to attempt to address aforementioned issues around the interim stopping rule in Gehan’s design being too relaxed, an extension to Gehan’s framework to make it more applicable to trials with high response rates has been presented [21]. We might hope a modification of this form may improve how the operating characteristics of Gehan’s design fair in comparison to Simon’s designs. However, in Additional file 1 we describe how a particular logical modification to the stage one stopping rule in Gehan’s design would be unlikely to result in improved statistical performance. Consequently, we believe it is also unlikely Gehan’s design will be preferable in situations where the response rate is anticipated to be large.

As we observed in Fig. 2, Gehan’s design is likely to have better performance in terms of the length of the final confidence interval when the response rate is much smaller than *π*_0_ and *π*_1_. However, this is simply a result of its increased requisite sample size. Furthermore, if *π*_0_ is known accurately based on reliable historical data, we would hope that this would be a rare occurrence. Ultimately, we feel that there is one principal situation in which Gehan’s designs may be particularly useful: when the primary goal of a trial is to estimate the response rate to a desired level of precision, and many patients are available to enroll in the study. This may occur perhaps when the regimen under investigation is a novel single-agent, in a more common cancer type. It was for this reason that we described design based on the functions *f*_*L*_ and *f*_*EL*_. With these, Gehan’s framework then provides a direct way to ensure that the response rate can be estimated precisely at the end of stage two. As, to guarantee the same precision with a two-stage group sequential design, a large search would need to be conducted over the possible design parameters to identify combinations that would lead to precise estimation on trial completion, across all possible true response rates. That is, the principal advantage in this setting would be computational. For, it may well be the case, as was evident for the example design utilising *f*_*L*_ in the previous section, that the required second stage sample sizes are constant for all *s*_1_, meaning the Gehan design functions in a similar manner to a group-sequential design. Of course, one should note that designs which provide such precise final estimates could require significantly increased sample sizes to those typically associated with single-arm phase II trials.

A useful compromise between the two competing designs could be to prospectively plan to use a flexible two-stage design [7]. With this, at the interim analysis, the remainder of the trial could then be specified in a group sequential design style, to retain the simplicity of Simon’s original designs. Alternatively, investigators could based on the interim data decide to take a Gehan like approach and complete stage two to achieve a precise final estimate of the response rate.

## Conclusions

We can readily incorporate a hypothesis test in to Gehan’s two-stage design, resolving one of its primary limitations. However, trialists should think carefully about using this design in practice, as Simon’s designs may often have advantageous or comparable performance in terms of their required sample size and the precision to which they will be able to estimate the response rate.

## Additional files

Additional file 1 Survey of studies utilising Gehan’s design and Design comparison based on Lorenzen et al. (2008). Details of how the survey to evaluate the number of studies that have utilised Gehan’s design was conducted are provided. In addition, an additional comparison of the performance of Gehan’s and Simon’s designs is given, based on the trial reported in Lorenzen et al. (2008) [17]. (PDF 181 kb)

Additional file 2 Survey results. An.xlsx file containing the results of the survey described in the Introduction and in Additional file 1. (XLSX 482 kb)

Additional file 3 R code. R code to determine the designs discussed in the manuscript and additional files, and reproduce each of the tables and figures. (R 36.6 kb)

Additional file 4 Julia code. Julia code to determine the confidence intervals for the designs discussed in the manuscript and additional files. (JL 11.7 kb)

Additional file 5 Confidence intervals. A.zip file containing.csv files that store the confidence intervals for the designs discussed in the manuscript and additional files. (ZIP 27.6 kb)

## Abbreviations

CEL: Conditional expected length
DCEF: Discrete conditional error function
ESS: Expected sample size
