# Supplementary material for: Re-formulating Gehan’s design as a flexible two-stage single-arm trial
Source: BMC Med Res Methodol. 2019 Jan 28;19:22. doi: 10.1186/s12874-019-0659-2 (PMC6350340; doi:10.1186/s12874-019-0659-2)
Supplement: Supplementary file 1 — Survey of studies utilising Gehan’s design and Design comparison based on Lorenzen et al. (2008). Details of how the survey to evaluate the number of studies that have utilised Gehan’s design was conducted are provided. In addition, an additional comparison of the performance of Gehan’s and Simon’s designs is given, based on the trial reported in Lorenzen et al. (2008) [17]. (PDF 181 kb) [file 12874_2019_659_MOESM1_ESM.pdf]

Additional file 1 for “Re-formulating Gehan’s design as a flexible two-stage single-arm trial” by Grayling and Mander

Survey of studies utilising Gehan’s design

In order to assess the number of times Gehan’s design has been used in published studies, we performed a survey of the articles that have cited his 1961 paper in the last ten years according to Google Scholar, and additionally searched and reviewed the PubMed Central articles containing “Gehan” over the same time period.

Firstly, Google Scholar (https://scholar.google.co.uk/) was used on September 30 2018 to identify records citing Gehan’s paper from January 1 2008 onwards (using the ‘Custom range’ field). Two hundred such records were acquired, which were exported first to Mendeley using the Mendeley Web Importer, and subsequently to a .csv file.

Similarly, PubMed Central was searched on September 30 2018 in order to identify any articles with a publication date of January 1 2008 or later that have contained “Gehan” in any field [search: (Gehan) AND (“2008/01/01”[Publication Date] : “2018/09/30”[Publication Date])]. One thousand eight hundred and seventy two records were extracted to an additional .csv file.

Each of the records was reviewed by MJG to determine which identified themselves as having used Gehan’s methodology, or a modified version there of. Where any confusion arose around the classification, a decision was made jointly with APM. We provide the results of our survey as Additional file 2.

Ultimately, two records acquired via Google Scholar were found to be duplicates of other citing articles, four to not be published articles (e.g., they were slides from a conference presentation), and one was excluded because its publication date fell outside of the considered range. We were unable to review 20 records; 11 because they were not written in English, and nine because we were unable to retrieve the full article. Of the other records, 52 were identified that used Gehan’s design or a modified Gehan design.

Additional results on the power of Gehan's design

Here, we provide the type-I error-rate, power, and values of $ESS(\pi_0)$ and $ESS(\pi_1)$ of the optimised Gehan designs, for additional parameter combinations. Precisely, in Table A1 the results correspond to $(\beta_1, \gamma, \pi_1) \in \{0.05, 0.1\} \times \{0.05, 0.1\} \times \{0.35, 0.4, 0.45, \dots, 0.7\}$ with $\pi_0 = \pi_1 - 0.15$. Similarly, Table A2 corresponds to $(\beta_1, \gamma, \pi_1) \in \{0.05, 0.1\} \times \{0.05, 0.1\} \times \{0.25, 0.3, 0.35, \dots, 0.7\}$ with $\pi_0 = \pi_1 - 0.2$, Table A3 to $(\beta_1, \gamma, \pi_1) \in \{0.05, 0.1\} \times \{0.05, 0.1\} \times \{0.3, 0.35, 0.4, \dots, 0.7\}$ with $\pi_0 = \pi_1 - 0.25$, and Table A4 to $(\beta_1, \gamma, \pi_1) \in \{0.05, 0.1\} \times \{0.05, 0.1\} \times \{0.35, 0.4, 0.45, \dots, 0.7\}$ with $\pi_0 = \pi_1 - 0.3$. In all cases, $\alpha = 0.05$, and results are provided for both the original and conservative methods for specifying $\hat{\pi}$ at the end of stage one in Gehan's original f_G.

We observe that when using the conservative approach to specifying $\hat{\pi}$, in no instance is the optimisation procedure unable to identify a design that controls the type-I error-rate to below 0.05. However, this is not the case for the original approach, through which the value of n_1 is frequently too small for a discrete conditional error function (DCEF) to exist which controls the type-I error-rate, particularly when π_0 is large.

In addition, as noted in the main manuscript, there are several instances in which $P(\pi_0) \ll \alpha$. There does not appear to be a clear pattern as to when this occurs, but it does happen more often with the original, rather than conservative, method for specifying $\hat{\pi}$ at the end of stage one.

Comparison of two-stage group sequential designs with $f_1 = 0$ to Gehan's design

In the main manuscript, we noted that Gehan's design may appear preferable to Simon's designs when the response rate is small, but that a non-optimal two-stage group sequential design may often exist that has similar performance. Here, we elaborate on this point. Consider again the parameters motivated by Dupuis-Girod *et al.* (2012); $\beta_1 = 0.1$, $\pi_0 = 0.15$, $\pi_1 = 0.3$, $\gamma = 0.1$, $\alpha = 0.05$, and $\beta = 0.2$.

Searching over two-stage group-sequential designs with a maximal allowed sample size of 100 patients, there are 114932 designs that meet the desired type-I error-rate and power. Of these, 10270 have $f_1 = 0$. Amongst these, that with the smallest sample size under H_0 (the 'null-optimal design with $f_1 = 0$'), and that with the smallest maximal sample size (the 'minimax design with $f_1 = 0$'), have the following design parameters

$$\text{Null-optimal design with } f_1 = 0: \ n_1 = 7, \ f_2 = 12, \ n_2 = 47,$$

$$\text{Minimax design with } f_1 = 0: \ n_1 = 10, \ f_2 = 11, \ n_2 = 38.$$

Table A1: Optimal hypothesis tests in Gehan designs using f_G. A summary of the type-I error-rate, $P(\pi_0)$, and power, $P(\pi_1)$, as well as the expected sample sizes when π_0 and π_1, $ESS(\pi_0)$ and $ESS(\pi_1)$, are shown for the optimal choices of the $D(s_1)$, for a range of values of β_1, γ, and π_1. In all cases, $\pi_0 = \pi_1 - 0.15$. Blank lines indicate a parameter and method combination for which no design exists that control the type-I error-rate to below the desired level of 0.05. All values are given to 3 decimal places.

			Original				Conservative			
β_1	γ	π_1	$P(\pi_0)$	$P(\pi_1)$	$ESS(\pi_0)$	$ESS(\pi_1)$	$P(\pi_0)$	$P(\pi_1)$	$ESS(\pi_0)$	$ESS(\pi_1)$
0.050	0.050	0.350	0.050	0.899	72.467	81.766	0.050	0.916	78.916	93.525
0.100	0.050	0.350	0.049	0.875	68.306	77.161	0.050	0.892	73.268	89.495
0.050	0.100	0.350	0.049	0.481	19.223	20.781	0.048	0.530	21.225	24.114
0.100	0.100	0.350	0.046	0.463	18.562	20.052	0.050	0.508	19.497	22.713
0.050	0.050	0.400	0.050	0.868	74.100	75.210	0.050	0.900	80.629	91.950
0.100	0.050	0.400	0.050	0.828	68.105	69.166	0.050	0.875	75.630	89.870
0.050	0.100	0.400	0.041	0.396	19.665	19.506	0.049	0.480	20.958	23.199
0.100	0.100	0.400	0.046	0.395	18.359	18.248	0.048	0.477	19.900	22.848
0.050	0.050	0.450	0.048	0.835	76.917	71.422	0.050	0.899	85.859	93.545
0.100	0.050	0.450					0.050	0.848	72.541	86.593
0.050	0.100	0.450	0.050	0.346	20.133	18.580	0.049	0.463	21.994	23.513
0.100	0.100	0.450					0.049	0.452	18.983	22.078
0.050	0.050	0.500					0.050	0.890	86.493	93.906
0.100	0.050	0.500					0.049	0.861	78.355	89.312
0.050	0.100	0.500					0.049	0.444	22.151	23.688
0.100	0.100	0.500					0.049	0.435	20.259	22.688
0.050	0.050	0.550					0.049	0.870	82.989	91.289
0.100	0.050	0.550					0.049	0.822	71.128	82.993
0.050	0.100	0.550					0.050	0.417	21.280	23.139
0.100	0.100	0.550					0.048	0.408	18.808	21.510
0.050	0.050	0.600					0.049	0.885	86.593	92.661
0.100	0.050	0.600					0.050	0.847	75.694	85.872
0.050	0.100	0.600					0.049	0.426	22.078	23.464
0.100	0.100	0.600					0.049	0.398	19.855	22.152
0.050	0.050	0.650					0.049	0.865	79.625	88.334
0.100	0.050	0.650					0.049	0.865	79.625	88.334
0.050	0.100	0.650					0.047	0.421	20.750	22.692
0.100	0.100	0.650					0.047	0.421	20.750	22.692

Table A2: Optimal hypothesis tests in Gehan designs using f_G. A summary of the type-I error-rate, $P(\pi_0)$, and power, $P(\pi_1)$, as well as the expected sample sizes when π_0 and π_1, $ESS(\pi_0)$ and $ESS(\pi_1)$, are shown for the optimal choices of the $D(s_1)$, for a range of values of β_1, γ, and π_1. In all cases, $\pi_0 = \pi_1 - 0.2$. Blank lines indicate a parameter and method combination for which no design exists that control the type-I error-rate to below the desired level of 0.05. All values are given to 3 decimal places.

			Original				Conservative			
β_1	γ	π_1	$P(\pi_0)$	$P(\pi_1)$	$ESS(\pi_0)$	$ESS(\pi_1)$	$P(\pi_0)$	$P(\pi_1)$	$ESS(\pi_0)$	$ESS(\pi_1)$
0.050	0.050	0.250	0.006	0.955	35.709	85.193	0.049	0.958	43.429	92.219
0.100	0.050	0.250	0.016	0.924	33.969	83.088	0.049	0.925	39.944	90.499
0.050	0.100	0.250	0.001	0.376	13.813	21.979	0.001	0.403	15.633	23.531
0.100	0.100	0.250	0.001	0.464	12.763	21.446	0.012	0.724	14.319	23.348
0.050	0.050	0.300	0.049	0.958	52.961	86.330	0.050	0.959	61.160	94.102
0.100	0.050	0.300	0.050	0.917	49.289	81.433	0.050	0.917	54.475	90.501
0.050	0.100	0.300	0.035	0.705	15.904	22.065	0.040	0.785	18.026	24.042
0.100	0.100	0.300	0.009	0.562	14.833	20.829	0.037	0.778	16.391	23.517
0.050	0.050	0.350	0.049	0.949	62.925	81.766	0.050	0.950	68.827	93.525
0.100	0.050	0.350	0.050	0.923	59.100	77.161	0.050	0.924	63.094	89.495
0.050	0.100	0.350	0.021	0.592	17.416	20.781	0.049	0.755	19.230	24.114
0.100	0.100	0.350	0.047	0.704	16.738	20.052	0.049	0.739	17.471	22.713
0.050	0.050	0.400	0.049	0.948	68.306	75.210	0.050	0.951	73.268	91.950
0.100	0.050	0.400	0.047	0.916	62.651	69.166	0.050	0.920	67.427	89.870
0.050	0.100	0.400	0.042	0.643	18.562	19.506	0.050	0.694	19.497	23.199
0.100	0.100	0.400	0.048	0.623	17.288	18.248	0.049	0.692	18.190	22.848
0.050	0.050	0.450	0.050	0.959	74.100	71.422	0.050	0.967	80.629	93.545
0.100	0.050	0.450					0.050	0.904	65.395	86.593
0.050	0.100	0.450	0.041	0.570	19.665	18.580	0.049	0.669	20.958	23.513
0.100	0.100	0.450					0.049	0.652	17.418	22.078
0.050	0.050	0.500	0.050	0.921	70.768	60.625	0.050	0.962	81.857	93.906
0.100	0.050	0.500					0.050	0.931	72.541	89.312
0.050	0.100	0.500	0.047	0.468	18.818	16.250	0.049	0.644	21.193	23.688
0.100	0.100	0.500					0.049	0.641	18.983	22.688
0.050	0.050	0.550					0.049	0.950	78.355	91.289
0.100	0.050	0.550	0.046	0.480	43.677	34.779	0.049	0.899	65.854	82.993
0.050	0.100	0.550					0.049	0.624	20.259	23.139
0.100	0.100	0.550					0.047	0.589	17.593	21.510
0.050	0.050	0.600					0.049	0.964	82.989	92.661
0.100	0.050	0.600					0.049	0.925	71.128	85.872
0.050	0.100	0.600					0.050	0.606	21.280	23.464
0.100	0.100	0.600					0.048	0.596	18.808	22.152
0.050	0.050	0.650					0.050	0.947	75.694	88.334
0.100	0.050	0.650					0.050	0.947	75.694	88.334
0.050	0.100	0.650					0.049	0.588	19.855	22.692
0.100	0.100	0.650					0.049	0.588	19.855	22.692
0.050	0.050	0.700					0.049	0.964	79.625	90.451
0.100	0.050	0.700					0.043	0.904	73.500	87.260
0.050	0.100	0.700					0.047	0.626	20.750	23.146
0.100	0.100	0.700					0.047	0.614	18.750	21.950

Table A3: Optimal hypothesis tests in Gehan designs using f_G. A summary of the type-I error-rate, $P(\pi_0)$, and power, $P(\pi_1)$, as well as the expected sample sizes when π_0 and π_1, $ESS(\pi_0)$ and $ESS(\pi_1)$, are shown for the optimal choices of the $D(s_1)$, for a range of values of β_1, γ, and π_1. In all cases, $\pi_0 = \pi_1 - 0.25$. Blank lines indicate a parameter and method combination for which no design exists that control the type-I error-rate to below the desired level of 0.05. All values are given to 3 decimal places.

			Original				Conservative			
β_1	γ	π_1	$P(\pi_0)$	$P(\pi_1)$	$ESS(\pi_0)$	$ESS(\pi_1)$	$P(\pi_0)$	$P(\pi_1)$	$ESS(\pi_0)$	$ESS(\pi_1)$
0.050	0.050	0.300	0.016	0.960	33.969	86.330	0.049	0.960	39.944	94.102
0.100	0.050	0.300	0.038	0.918	30.903	81.433	0.045	0.918	34.451	90.501
0.050	0.100	0.300	0.001	0.663	12.763	22.065	0.012	0.855	14.319	24.042
0.100	0.100	0.300	0.002	0.728	11.392	20.829	0.025	0.893	12.430	23.517
0.050	0.050	0.350	0.050	0.951	49.289	81.766	0.050	0.951	54.475	93.525
0.100	0.050	0.350	0.049	0.925	46.004	77.161	0.050	0.925	49.247	89.495
0.050	0.100	0.350	0.009	0.723	14.833	20.781	0.037	0.891	16.391	24.114
0.100	0.100	0.350	0.043	0.843	14.102	20.052	0.041	0.873	14.703	22.713
0.050	0.050	0.400	0.049	0.953	59.100	75.210	0.049	0.953	63.094	91.950
0.100	0.050	0.400	0.048	0.922	54.023	69.166	0.050	0.922	56.803	89.870
0.050	0.100	0.400	0.047	0.830	16.738	19.506	0.049	0.864	17.471	23.199
0.100	0.100	0.400	0.045	0.821	15.512	18.248	0.050	0.833	15.963	22.848
0.050	0.050	0.450	0.049	0.972	68.306	71.422	0.050	0.972	73.268	93.545
0.100	0.050	0.450	0.049	0.908	52.998	55.904	0.050	0.908	56.757	86.593
0.050	0.100	0.450	0.042	0.792	18.562	18.580	0.050	0.837	19.497	23.513
0.100	0.100	0.450	0.048	0.723	14.598	15.067	0.050	0.806	15.528	22.078
0.050	0.050	0.500	0.049	0.967	68.105	60.625	0.050	0.969	75.630	93.906
0.100	0.050	0.500					0.050	0.937	65.395	89.312
0.050	0.100	0.500	0.046	0.716	18.359	16.250	0.048	0.819	19.900	23.688
0.100	0.100	0.500					0.049	0.799	17.418	22.688
0.050	0.050	0.550					0.050	0.959	72.541	91.289
0.100	0.050	0.550	0.043	0.604	43.194	34.779	0.050	0.909	59.799	82.993
0.050	0.100	0.550					0.049	0.798	18.983	23.139
0.100	0.100	0.550					0.048	0.762	16.194	21.510
0.050	0.050	0.600					0.049	0.974	78.355	92.661
0.100	0.050	0.600	0.046	0.533	43.677	30.792	0.049	0.935	65.854	85.872
0.050	0.100	0.600					0.049	0.788	20.259	23.464
0.100	0.100	0.600					0.047	0.753	17.593	22.152
0.050	0.050	0.650					0.049	0.957	71.128	88.334
0.100	0.050	0.650					0.049	0.957	71.128	88.334
0.050	0.100	0.650					0.048	0.766	18.808	22.692
0.100	0.100	0.650					0.048	0.766	18.808	22.692
0.050	0.050	0.700					0.050	0.973	75.694	90.451
0.100	0.050	0.700					0.043	0.910	68.735	87.260
0.050	0.100	0.700					0.040	0.767	19.855	23.146
0.100	0.100	0.700					0.039	0.741	17.637	21.950

Table A4: Optimal hypothesis tests in Gehan designs using f_G. A summary of the type-I error-rate, $P(\pi_0)$, and power, $P(\pi_1)$, as well as the expected sample sizes when π_0 and π_1, $ESS(\pi_0)$ and $ESS(\pi_1)$, are shown for the optimal choices of the $D(s_1)$, for a range of values of β_1, γ, and π_1. In all cases, $\pi_0 = \pi_1 - 0.3$. Blank lines indicate a parameter and method combination for which no design exists that control the type-I error-rate to below the desired level of 0.05. All values are given to 3 decimal places.

			Original				Conservative			
β_1	γ	π_1	$P(\pi_0)$	$P(\pi_1)$	$ESS(\pi_0)$	$ESS(\pi_1)$	$P(\pi_0)$	$P(\pi_1)$	$ESS(\pi_0)$	$ESS(\pi_1)$
0.050	0.050	0.350	0.038	0.951	30.903	81.766	0.045	0.951	34.451	93.525
0.100	0.050	0.350	0.046	0.925	28.497	77.161	0.050	0.925	30.656	89.495
0.050	0.100	0.350	0.002	0.843	11.392	20.781	0.025	0.943	12.430	24.114
0.100	0.100	0.350	0.014	0.889	10.557	20.052	0.005	0.895	10.972	22.713
0.050	0.050	0.400	0.049	0.953	46.004	75.210	0.050	0.953	49.247	91.950
0.100	0.050	0.400	0.050	0.922	41.823	69.166	0.050	0.922	43.251	89.870
0.050	0.100	0.400	0.043	0.909	14.102	19.506	0.041	0.935	14.703	23.199
0.100	0.100	0.400	0.039	0.902	12.938	18.248	0.041	0.907	13.109	22.848
0.050	0.050	0.450	0.049	0.972	59.100	71.422	0.049	0.972	63.094	93.545
0.100	0.050	0.450	0.044	0.908	45.518	55.904	0.050	0.908	46.466	86.593
0.050	0.100	0.450	0.040	0.909	16.738	18.580	0.049	0.937	17.471	23.513
0.100	0.100	0.450	0.034	0.845	13.006	15.067	0.049	0.881	13.278	22.078
0.050	0.050	0.500	0.048	0.969	62.651	60.625	0.050	0.969	67.427	93.906
0.100	0.050	0.500	0.049	0.937	52.998	51.625	0.050	0.937	56.757	89.312
0.050	0.100	0.500	0.047	0.881	17.288	16.250	0.049	0.919	18.190	23.688
0.100	0.100	0.500	0.048	0.840	14.598	14.125	0.050	0.894	15.528	22.688
0.050	0.050	0.550					0.050	0.959	65.395	91.289
0.100	0.050	0.550	0.048	0.743	41.250	34.779	0.050	0.909	52.891	82.993
0.050	0.100	0.550					0.049	0.898	17.418	23.139
0.100	0.100	0.550					0.047	0.841	14.594	21.510
0.050	0.050	0.600					0.050	0.974	72.541	92.661
0.100	0.050	0.600	0.043	0.650	43.194	30.792	0.050	0.936	59.799	85.872
0.050	0.100	0.600					0.049	0.904	18.983	23.464
0.100	0.100	0.600					0.048	0.870	16.194	22.152
0.050	0.050	0.650	0.046	0.565	43.677	26.478	0.049	0.957	65.854	88.334
0.100	0.050	0.650	0.046	0.565	43.677	26.478	0.049	0.957	65.854	88.334
0.050	0.100	0.650					0.047	0.872	17.593	22.692
0.100	0.100	0.650					0.047	0.872	17.593	22.692
0.050	0.050	0.700					0.049	0.973	71.128	90.451
0.100	0.050	0.700					0.048	0.910	63.440	87.260
0.050	0.100	0.700					0.048	0.888	18.808	23.146
0.100	0.100	0.700					0.045	0.847	16.400	21.950

Figure A1 depicts the expected sample size (ESS) curves of the above designs, along with the optimised Gehan designs presented in the main manuscript. Figure A2 contains the conditional expected length (CEL) curves of the same four designs. We can see that, as noted, it is difficult in this instance to argue that either of the Gehan designs should be preferred.

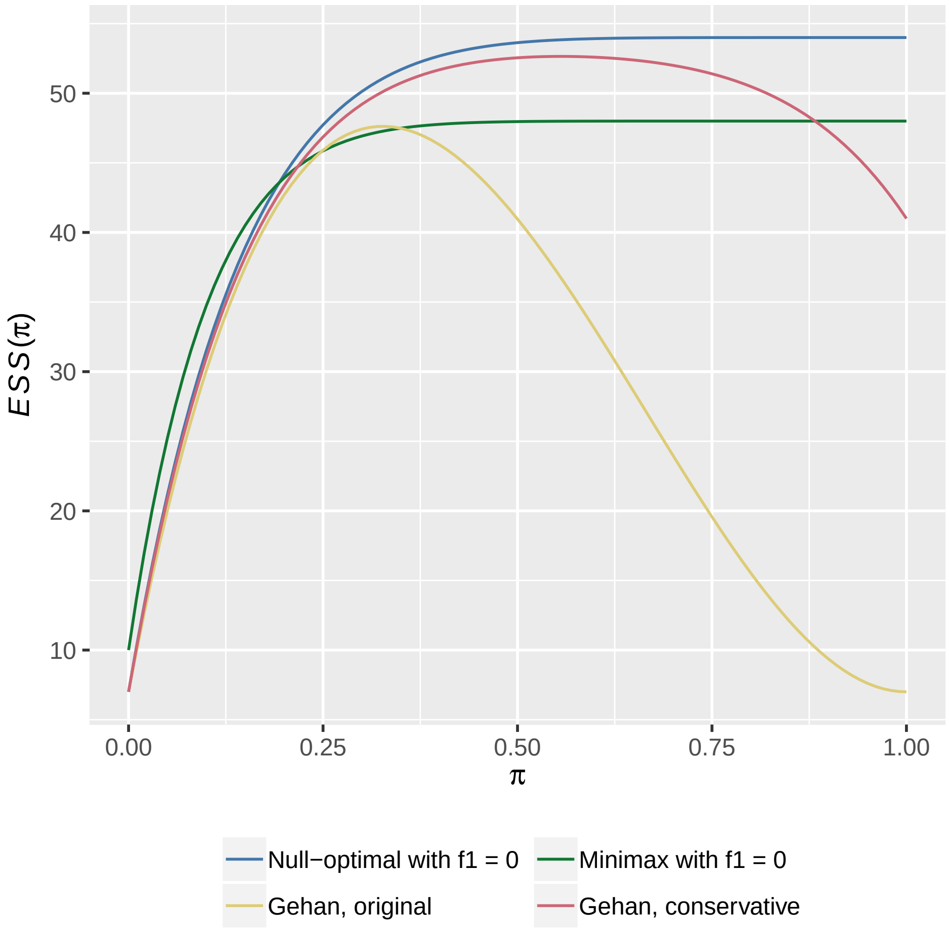

Figure A1: Shows the $ESS(\pi)$ curves for Gehan's designs using the original and conservative methods for specifying $\hat{\pi}$ in f_G, and Simon's null-optimal and minimax designs with $f_1 = 0$.

Modifying the interim stopping rule in Gehan's design

In this section, we describe a logical method by which the interim stopping rule in Gehan's design could be modified in order to decrease the probability stage two is commenced. One may hope that this would increase the efficiency of the design when π_0 and π_1 are large.

We implement a stopping rule of $S_1 \leq f_1$, with f_1 a function of n_1 and π_0, which we denote by $f_1(n_1)$. Precisely, a value α_1 is specified, and then $f_1(n_1)$ is chosen as

$$\underset{f_1 \in \mathbb{N}^+}{\text{argmin}}\{\mathbb{P}(S_1 > f_1 | n_1, \pi_0) \leq \alpha_1\}, \tag{0.1}$$

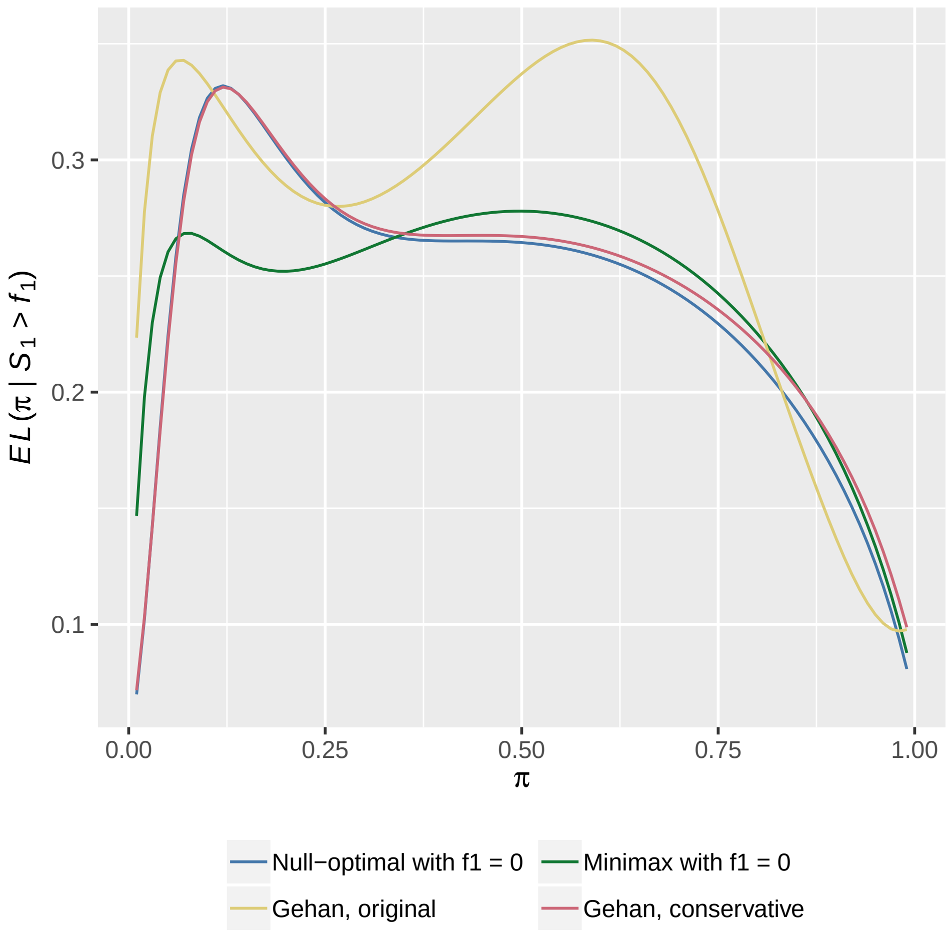

Figure A2: Shows the $EL(\pi \mid S_1 > f_1)$ curves for Gehan's designs using the original and conservative methods for specifying $\hat{\pi}$ in f_G, and Simon's null-optimal and minimax designs with $f_1 = 0$.

where $\mathbb{P}(S_1 > f_1|n_1, \pi_0) = 1 - B(f_1|n_1, \pi_0)$. That is, f_1 is chosen to ensure the probability stage-two is commenced when $\pi = \pi_0$ is at most α_1. Then, n_1 can be chosen as the solution to

$$\underset{n_1 \in \mathbb{N}^+}{\operatorname{argmin}}[\mathbb{P}\{S_1 \le f_1(n_1)|n_1, \pi_1\} \le \beta_1], \tag{0.2}$$

and the $n_2(s_1)$ and the $D(s_1)$ optimised in the same manner as before. In practice, α_1 could be chosen based upon the probability of early termination in a corresponding Simon two-stage design. Note that setting $\alpha_1 \ge 1$ reduces the design to Gehan's original proposal.

As was noted, however, the problem with this approach in practice is that as α_1 is decreased the resulting value of n_1 will increase. Consequently, the overall efficiency of the design may not improve. We demonstrate this here for an example with $\pi_0 = 0.3$, $\pi_1 = 0.5$, $\beta_1 = 0.05$, $\gamma = 0.1$, and $\alpha = 0.1$. We identified the optimised Gehan designs for $\alpha_1 \in \{1, 0.8, 0.7, 0.6, 0.575, 0.5, 0.45, 0.4\}$, which result in the unique possible values of f_1. The ESS and CEL curves of these designs are given in Supplementary Figures A3 and A4 respectively. As can be seen, the performance of the original design with $f_1 = 0$, is not dissimilar to those with $f_1 \in \{1, 2, 3\}$.

Note that an attempt to rectify the above by increasing f_1 whilst holding n_1 constant is unlikely to be useful, as the power of the resulting design would be expected to drop markedly.

Design comparison based on Lorenzen et al. (2008)

Here, we focus on design for our motivating scenario based on Lorenzen *et al.* (2008), for which $\beta_1 = 0.05$, $\pi_1 = 0.3$, $\pi_1 = 0.5$, and $\gamma = 0.1$. In this case, our optimal version of Gehan's design when using the original approach to specfiying $\hat{\pi}$ in f_G has $n_1 = 5$ and

$$\begin{aligned}
D(0) = 0,\ D(1) = 0.0480,\ D(2) = 0.0596,\ D(3) = 0.1941,\ D(4) = D(5) = 1, \\
n_2(0) = 0,\ n_2(1) = 20,\ n_2(2) = 18,\ n_2(3) = 8,\ n_2(4) = n_2(5) = 0, \\
P(\pi_0) = 0.092,\ P(\pi_1) = 0.664, \\
ESS(\pi_0) = 18.82,\ ESS(\pi_1) = 16.25.
\end{aligned}$$

Using the conservative approach instead, we find

$$\begin{aligned}
D(0) = 0,\ D(1) = 0.0480,\ D(2) = 0.0839,\ D(3) = 0.3345,\ D(4) = 0.3920,\ D(5) = 0.4656, \\
n_2(0) = 0,\ n_2(1) = 20,\ n_2(2) = n_2(3) = 19,\ n_2(4) = 20,\ n_2(5) = 18, \\
P(\pi_0) = 0.100,\ P(\pi_1) = 0.767, \\
ESS(\pi_0) = 21.19,\ ESS(\pi_1) = 23.69.
\end{aligned}$$

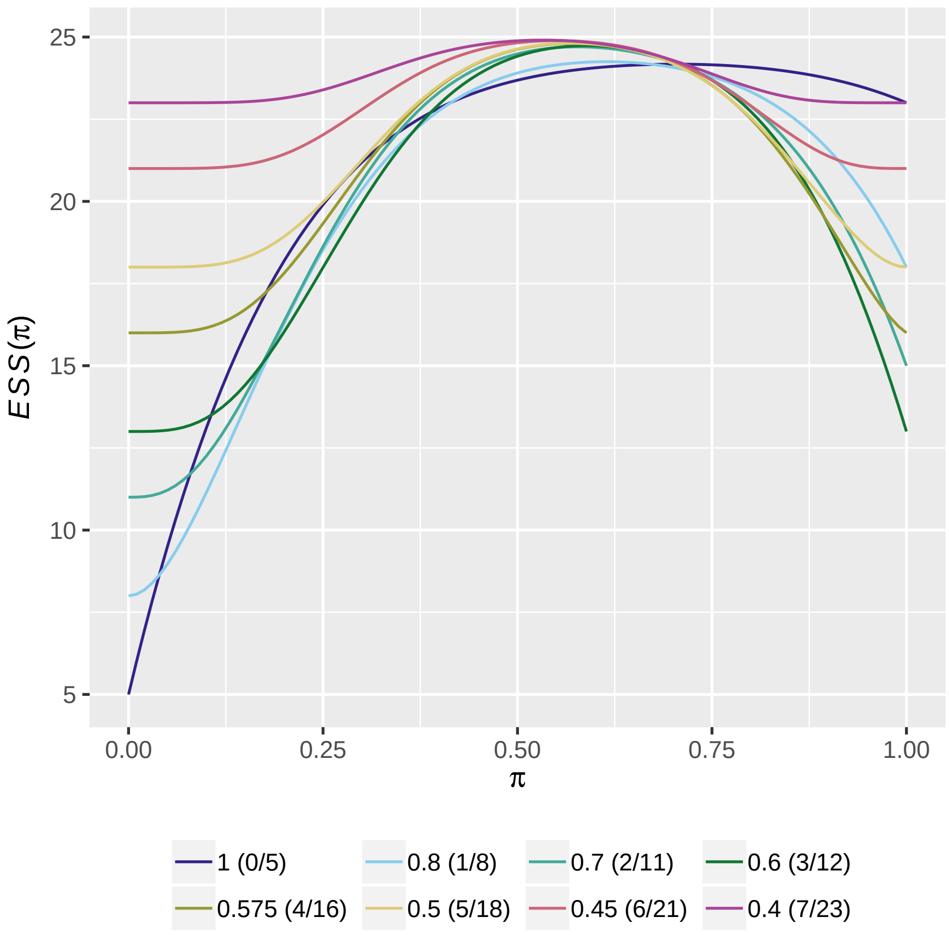

Figure A3: Shows the $ESS(\pi)$ curves for Gehan's designs using the conservative method for specifying $\hat{\pi}$ in f_G, with differing values of α_1. The lines are labelled in the form α_1 (f_1/n_1).

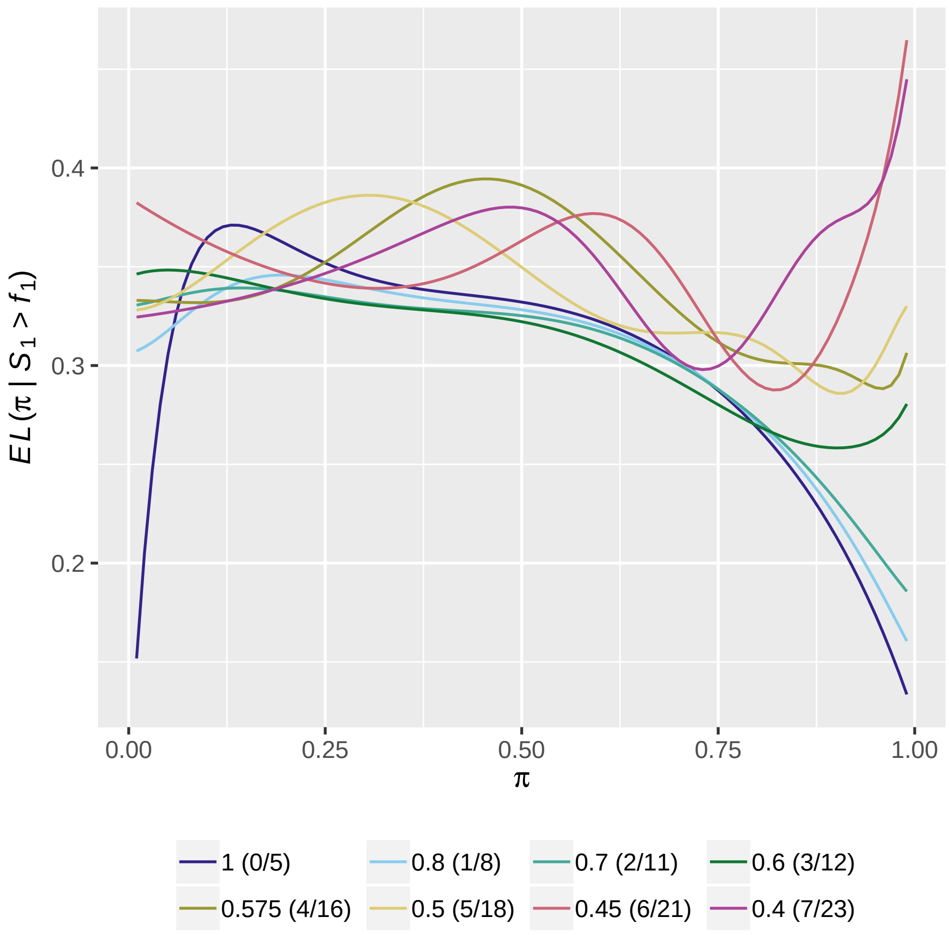

Figure A4: Shows the $EL(\pi \mid S_1 > f_1)$ curves for Gehan's designs using the conservative method for specifying $\hat{\pi}$ in f_G, with differing values of α_1. The lines are labelled in the form α_1 (f_1/n_1).

Suppose that once more we desire 80% power, then these modified Gehan designs are again unable to provide that required. Therefore, as in the main manuscript, we search for the maximal γ such that $P(\pi_1) \geq 0.8$. Completing this search, for the original method, we find that $\gamma = 0.0805$ gives a design with $n_1 = 5$ and

$$\begin{aligned}
D(0) &= 0,\ D(1) = 0.0437,\ D(2) = 0.0534,\ D(3) = 0.2784,\ D(4) = D(5) = 1,\\
n_2(0) &= 0,\ n_2(1) = 33,\ n_2(2) = 31,\ n_2(3) = 15,\ n_2(4) = n_2(5) = 0,\\
P(\pi_0) &= 0.099,\ P(\pi_1) = 0.810,\\
ESS(\pi_0) &= 28.44,\ ESS(\pi_1) = 24.53.
\end{aligned}$$

Whilst for the conservative method we find that $\gamma = 0.0943$ gives a design with $n_1 = 5$ and

$$\begin{aligned}
D(0) &= 0,\ D(1) = 0.0546,\ D(2) = 0.1201,\ D(3) = 0.2291,\ D(4) = 0.3819,\ D(5) = 0.8016,\\
n_2(0) &= 0,\ n_2(1) = n_2(2) = n_2(3) = n_2(4) = 23,\ n_2(5) = 21,\\
P(\pi_0) &= 0.099,\ P(\pi_1) = 0.812,\\
ESS(\pi_0) &= 24.13,\ ESS(\pi_1) = 27.22.
\end{aligned}$$

We now assess whether the optimal Gehan designs have better statistical characteristics than Simon's designs. Therefore, note that in this case Simon's designs are

$$\begin{aligned}
\text{Null-optimal}: &\ f_1 = 5,\ n_1 = 15,\ f_2 = 12,\ n_2 = 17,\\
\text{Minimax}: &\ f_1 = 3,\ n_1 = 12,\ f_2 = 11,\ n_2 = 16.
\end{aligned}$$

Thus, the conservative approach based Gehan design actually has a smaller maximal sample size than Simon's null-optimal design, and the same maximal sample size as the minimax design. To examine the required sample sizes of these designs further, we present their ESS curves in Figure A5. Here, the Gehan designs are again more efficient at low values of π because of their smaller value of n_1. Moreover, the Gehan design based on the original approach is the most efficient when $\pi = \pi_1$. However, there is a large region around $\pi = 0.3$ in which the Gehan designs are expected to require a substantially larger number of participants.

Our next consideration is again whether the Gehan designs estimate π to a better precision than Simon's designs when we do not stop for futility at the end of stage one. In Figure A6 we therefore compare the four CEL curves. Here, the CEL curve for the Gehan design with the conservative approach is always below the curves for Simon's designs. Thus, in the case where we anticipate a high response rate, when this Gehan design is expected to required fewer patients, it may be considered better than

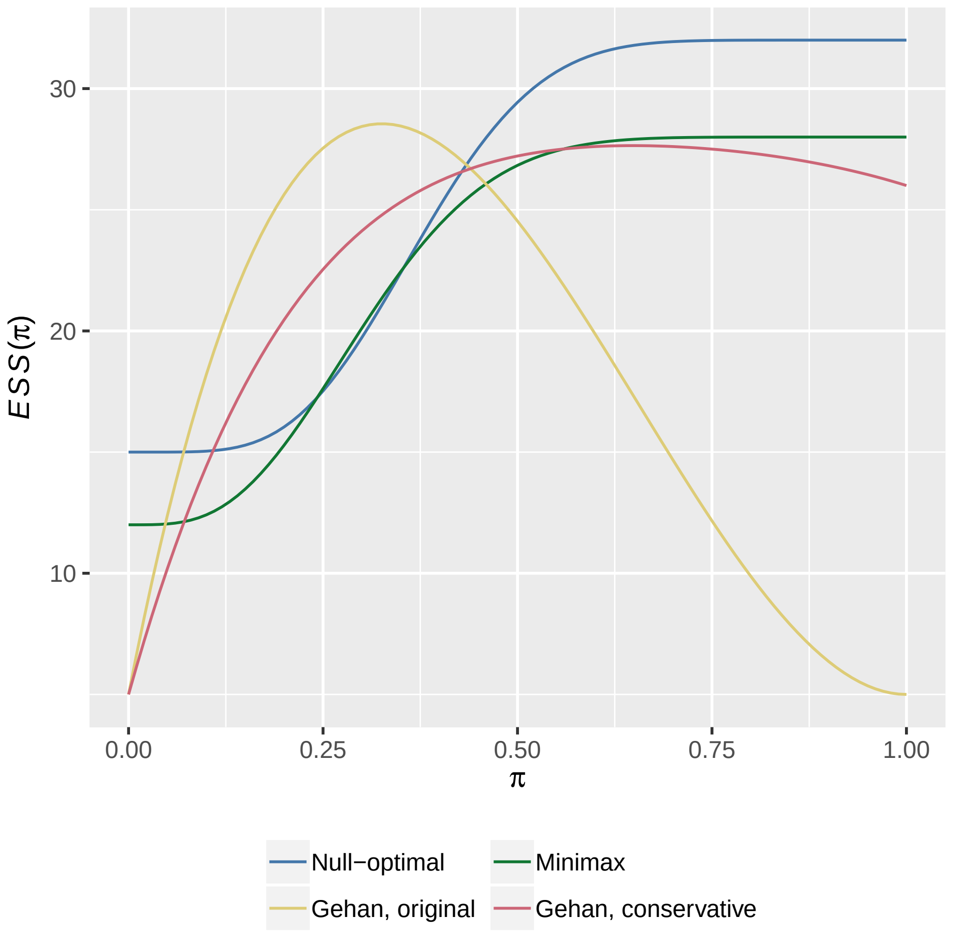

Figure A5: Shows the $ESS(\pi)$ curves for Gehan's designs using the original and conservative methods for specifying $\hat{\pi}$ in f_G, and Simon's null-optimal and minimax designs.

the null-optimal and minimax Simon designs.

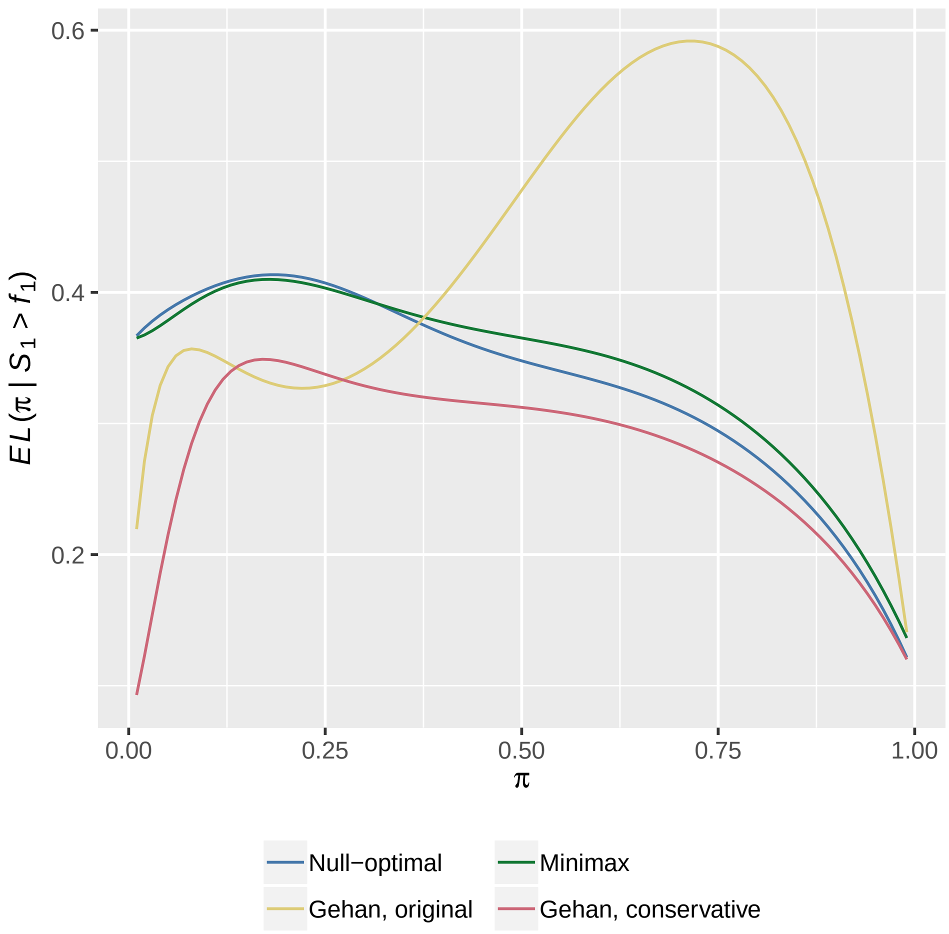

Figure A6: Shows the $EL(\pi \mid S_1 > f_1)$ curves for Gehan's designs using the original and conservative methods for specifying $\hat{\pi}$ in f_G, and Simon's null-optimal and minimax designs.
